# A Prediction Model of Disease Progression in X-Linked Alport syndrome Based on Clinical Characteristics and Genetic Variants

**DOI:** 10.1016/j.ekir.2025.03.006

**Published:** 2025-03-10

**Authors:** Mengyao Zeng, Hongling Di, Jie Ding, Yanqin Zhang, Hong Xu, Jingyuan Xie, Jianhua Mao, Aihua Zhang, Guisen Li, Jiahui Zhang, Erzhi Gao, Dandan Liang, Qing Wang, Ling Wang, Yu An, Chunxia Zheng, Zhihong Liu

**Affiliations:** 1 National Clinical Research Center of Kidney Diseases, Jinling Hospital, Medical School of Nanjing University, Nanjing, China; 2 Department of Pediatrics, Peking University First Hospital, Beijing, China; 3 Department of Nephrology, Children's Hospital of Fudan University, Shanghai, China; 4 Department of Nephrology, Shanghai Ruijin Hospital, Shanghai, China; 5 National Clinical Research Center for Child Health, the Children's Hospital, Zhejiang University School of Medicine, Hangzhou, China; 6 Department of Nephrology, Children's Hospital of Nanjing Medical University, Nanjing, China; 7 Department of Nephrology, Sichuan Provincial People's Hospital, Chengdu, China

**Keywords:** genetic variants, kidney failure, prediction model, risk stratification, X-linked Alport syndrome

## Abstract

**Introduction:**

Alport syndrome (AS) is an inherited kidney disease with significant clinical heterogeneity. Prognosis prediction and risk assessment are important to assist patient care. However, a predictive tool of disease progression is still lacking.

**Methods:**

The prediction model was developed in 363 patients (124 kidney failure events) with X-linked AS (XLAS) from a single-center retrospective cohort study and validated in 2 external cohorts, including 193 (27 events) and 125 patients (33 events) with XLAS from 6 centers and the literature database, respectively. Cox proportional hazards regression analysis with stepwise selection was used to select the important variables related to the progression to kidney failure, by using the baseline demographic, clinical, and genetic data. The performance of the prediction model was evaluated and compared using receiver-operating characteristic (ROC) curve and calibration plot.

**Results:**

There were 4 variables identified that were significantly associated with the progression to kidney failure in the final model, namely sex, proteinuria, estimated glomerular filtration rate (eGFR), and pathogenic variants in *COL4A5.* Based on the model risk stratification, the median age at kidney failure was 23, 30, and 61 years in the low-, intermediate-, and high-risk groups, respectively. This model shows the best discrimination in predicting the progression to kidney failure before the age of 30 years, with areas under the curve (AUCs) > 0.80 in both development and external cohorts.

**Conclusion:**

A prediction model of progression to kidney failure based on clinical characteristics and genetic variants was developed and validated in patients with XLAS.

## Graphical abstract

**Figure:**
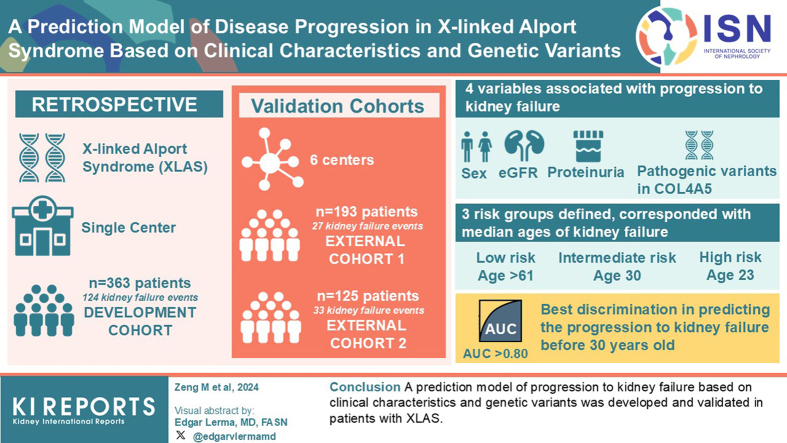

AS is an inherited kidney disease caused by pathogenic variants in any of the collagen IV genes *COL4A3*, *COL4A4*, *COL4A5*, and characterized by hematuria and progressive kidney failure, often with sensorineural hearing loss and ocular anomalies.^1^^,^^2^ AS presents with a wide spectrum of clinical presentations, ranging from isolated hematuria to kidney failure, according to the genetic types and sex. XLAS is the most frequent pattern of AS, which is caused by pathogenic variants in *COL4A5*. In patients with XLAS, males often exhibit a more severe phenotype than females. As reported, the median age of kidney failure in male XLAS is approximately 25 years; however, in females, 16% of the patients reach kidney failure by the age of 65 years.^3^^,^^4^ Pathogenic variants in *COL4A3* and *COL4A4* are associated with autosomal AS, which is divided into autosomal recessive AS (ARAS) and autosomal dominant AS (ADAS).^5^ Compared with XLAS and ARAS cases, patients with ADAS who are harboring a heterozygous pathogenic variant in *COL4A3* or *COL4A4* often exhibit milder phenotypes, with rare extrarenal features and a median kidney survival of 67 years.^6^ As a genetic disease with significant clinical heterogeneity, prognosis prediction and risk assessment in AS are important to guide clinical care, which may also be helpful to inform and reassure patients with AS about the anticipated course of their disease. However, a predictive tool for disease progression is still lacking for patients with AS, possibly limited by past inefficient genetic sequencing technologies and the small sample size or long course of this rare disease.

Based on next-generation sequencing technology, many studies have provided evidence that there are strong genotype-phenotype correlations in XLAS and ARAS. For example, patients harboring protein-truncating pathogenic variants would progress to kidney failure more quickly than patients with non truncating pathogenic variants in male XLAS, which may be applied for risk stratification of the disease.^7^, ^8^, ^9^ Besides genetic variants, there are many clinical or pathological factors related to disease progression of XLAS, such as proteinuria, hearing loss, and *α*5 expression on the glomerular basement membrane.^6^ As previous studies reported, there was a significant difference in renal prognosis between patients with XLAS with hearing loss or not (respective median renal survival periods of 28 and 55 years),^7^ and the presence of proteinuria significantly increased the risk of kidney failure in patients with ADAS, with a high hazard ratio (HR) of 5.57.^6^

In this study, we integrated genetic and clinical data from 363 patients with XLAS with long-term follow-up, aiming to evaluate the predictive value of clinical characteristics and genetic variants related to disease progression and to develop a prediction model with arisk-scoring system to forecast the progression to kidney failure in patients with XLAS.

## Methods

### Study Design and Participants

A single-center, retrospective cohort study was conducted to develop a prediction model. The data were from 363 patients with XLAS in the Renal Biopsy Registry of the National Clinical Research Center of Kidney Diseases at Jinling Hospital from 2003 to 2022. The eligibility criteria for inclusion were as follows: patients with typical glomerular basement membrane ultrastructural alterations and/or abnormal *α*3*α*4*α*5(IV) collagen chain staining confirmed by renal biopsy, and who carried a proven pathogenic *COL4A5* gene variant by genetic testing. Digenic cases were not included in our cohort.

Baseline clinical data of the patients were obtained during their first clinic visit, and the outcome information was collected during their follow-ups, both from the Renal Biopsy Registry. All follow-up data were updated to May 2023. The blood samples of patients collected during their first visit were genotyped with a consistent approach during their visits.

Two external cohorts were used to validate the performance of the prediction model. The multicenter cohort included 193 cases with XLAS from 6 other hospitals in China, who had complete information on the basic characteristics related to the progression to kidney failure, including sex, baseline proteinuria and serum creatinine at the first clinic visit, pathogenic variants in *COL4A5,* and age at the onset of kidney failure or the latest follow-up. Similarly, the database cohort contained 125 Chinese patients with XLAS with the complete information mentioned above, retrieved from the literature search of PubMed, Web of Science, and China National Knowledge Infrastructure. All sources were searched from inception to June 9, 2023. Their clinical data and genetic results were collected by clinicians using a standardized questionnaire.

The study was approved by the ethics committee of Jinling Hospital (2022DZKY-098-01). Written informed consent was obtained from all patients.

### Procedures

Baseline data of patients were obtained during their first clinic visit for AS to the Jinling hospital, including demographic information (sex and age), physical examination (height, weight, and blood pressure), laboratory testing (presence of hematuria, proteinuria, serum creatinine, fasting blood glucose, uric acid, triglyceride, and total cholesterol), extrarenal manifestations (ocular lesions and hearing loss) and lifestyle habits (smoking and alcohol consumption). Renal biopsies (if possible) with blood samples of the patients were also collected and stored during their first visit. Renal biopsy specimens were routinely processed using standard light, immunofluorescence, and electron microscopy procedures.^10^^,^^11^ Data about pathological features, such as *α*5 expression on glomerular basement membrane and the severity of the glomerular basement membrane ultrastructural lesions were recorded by pathologists. Once diagnosed, patients were followed-up with every 3 to 6 months through interviews to check for their disease progression and medication situation. The age at the onset of kidney failure and the use of angiotensin-converting enzyme inhibitor (ACEI) and/or angiotensin II receptor blocker (ARB) treatment of the patients were documented during the follow-ups.

Cases with ACEI or ARB treatment were defined as patients who were continuously exposed to ACEI or ARB treatment for ≥ 3 months and maintained during the follow-up period, whereas patients who never received ACEI or ARB or discontinued this treatment after a brief exposure of < 3 months were defined as without ACEI or ARB treatment. The most commonly used ACEIs were benazepril (10 mg/d) or perindopril (4–8 mg/d) and the most commonly used ARBs were losartan (50–100 mg/d) or valsartan (80 mg/d), as described in the previous study.^8^

### Definitions of Clinical Variables

The eGFR was calculated according to the Chronic Kidney Disease Epidemiology Collaborative equation.^12^ Hypertension was defined as a systolic blood pressure ≥ 140 mm Hg and/or diastolic blood pressure ≥ 90 mm Hg at the first clinic visit for AS, or if the patient had been diagnosed with definite hypertension.^13^^,^^14^ Hyperlipemia was defined as a total cholesterol concentration > 240 mg/dl and/or triglyceride concentration > 200 mg/dl.^15^, ^16^, ^17^ Hyperuricemia was defined as uric acid ≥ 7.0 mg/dl in men and ≥ 6.0 mg/dl in women.^18^ Body mass index was calculated and categorized into 3 groups according to the Working Group of China definition (< 18.5, 18.5–23.9, or ≥ 24.0 kg/m^2^).^19^

### Genetic Data

Genetic testing was conducted on both the test set and validation set cases by whole-exome sequencing, whole-genome sequencing, or a nephropathy gene panel containing the *COL4A3*, *COL4A4*, and *COL4A5* genes. Variant filtering and pathogenicity assessments were performed as previously described.^8^ Briefly, the heterozygous variants with minor allele frequency < 0.01% and the homozygotes or compound heterozygotes variants with minor allele frequency < 0.1% in the *COL4A5* gene were screened based on the gnomAD and ExAC databases. Then we predicted the pathogenicity of missense variants by using SIFT, PolyPhen-2, and CADD; and predicted the pathogenicity of splicing variants by using spliceAI and human splicing finder. Pathogenic variants in *COL4A5* were divided into 5 categories as follows: missense, splicing, nonsense, small insertion-deletions (indels), and copy number variation. Small indels are genomic alterations involving the gain or loss of DNA segments spanning 1 to 100 base pairs. These variants were classified into 2 functional categories as follows: in-frame indels (preserving the original triplet codon reading frame) and frameshift indels (shifting the translational reading frame), depending on whether the indel length is divisible by 3 nucleotides.^20^ By transcript type, pathogenic variants were also classified as protein-truncating variants and nontruncating variants.

### Statistical Analysis

Categorical baseline characteristics were reported as counts and percentages, whereas continuous variables were expressed as mean ± SD or median with interquartile range (IQR). Cox proportional hazards regression analysis was used to examine the univariate associations of candidate prognostic variables with the onset of kidney failure. Kaplan-Meier method with log-rank test was used to analyze kidney survival in different subgroups on the baseline characteristics. Variables associated with the progression to kidney failure at the *P* < 0.10 level were entered into the multivariate Cox proportional hazards regression. Two prediction models were developed; one is the clinical plus genetic model, which integrated genetic and clinical data; and the other one is the clinical-only model. Stepwise selection, with conservative entry (*P* = 0.05) and removal (*P* = 0.1) thresholds, was used to choose the independent variables that were retained in the models. The proportional hazard assumption was tested for each variable in the final models by using scaled Schoenfeld residuals.^21^

Based on the final prediction models, scoring systems were constructed. Weighted scores were assigned to the prognostic variables according to the regression coefficient in Cox proportional hazards regression analysis. The scores of each factor were obtained by dividing each HR by the smallest HR considered and rounding the resultant ratio to the integer in 2 models.^22^ Ten-fold cross-validation of the entire model-building process was conducted for internal validation. In this procedure, 10 random samples comprising 90% of the data were used to develop the model with the concatenation of the remaining 10% per fold used to evaluate model fit. Sex-stratified prediction models were also developed to examine the prognostic variables related to kidney failure in male and female XLAS.

Kaplan-Meier method and Cox proportional hazards regression were used to compare the prognostic kidney outcomes in patients with different risks. To assess the predictive performance of the prognostic models, the sensitivity, specificity, and positive and negative predictive values were estimated at several given cutoff values of ages at the onset of kidney failure, for different score threshold values by logistic regression in the development cohort and external cohorts. The performances of the prediction models were evaluated and compared by using ROC curves and calibration plots. Subgroup analysis was used to examine the prognostic kidney outcomes in patients with different risks among different subgroups on the baseline characteristics.

All statistical analyses were performed using the SAS software (version 9.4, SAS Institute, Cary, NC). A 2-tailed level of *P* < 0.05 was considered statistically significant.

## Results

### Study Cohorts

Of the 363 patients with XLAS included in the study, the median age of diagnosis was 21 (IQR: 16–27) years. After a median follow-up of 10 (IQR: 6–14) years, 124 patients (34%) had reached kidney failure. The baseline characteristics of the development and the other 2 external cohorts were shown in Table 1. We can find that the multicenter cohort was much younger, with a median age of 8 (IQR: 4–17) years and a follow-up of 3 (IQR: 0.5–5) years. The rates of kidney failure in the multicenter cohort and database cohort were 14% and 26%, respectively. Regarding the types of pathogenic variants, among the 47 patients with indels in the development cohort, 9 had in-frame indels and 38 had frameshift indels.

**Table 1:** Patient characteristics in the study cohorts

Characteristics	Development cohort	Multicenter cohort (external)	Database cohort (external)
Number of participants	363	193	125
Age, yrs	21 (16–27)	8 (4–17)	/
Follow-up period, yrs	10 (6–14)	3 (0.5–5)	/
Kidney failure events	124 (34)	27 (14)	33 (26)
Sex			
Male	254 (70)	133 (69)	73 (58)
Female	109 (30)	60 (31)	52 (42)
Baseline proteinuria, g/24 h			
< 0.4	30 (8)	81 (42)	28 (22)
0.4–3.4	247 (68)	82 (42)	56 (45)
≥ 3.5	86 (24)	30 (16)	41 (33)
Baseline eGFR, ml/min per 1.73 m^2^			
≥ 60	305 (84)	171 (89)	96 (77)
< 60	58 (16)	22 (11)	29 (23)
Hearing loss			
No	189 (60)	82 (59.4)	28 (47)
Yes	124 (40)	56 (40.6)	32 (53)
Missing	50	55	65
Type of pathogenic variants			
Missense	222 (61)	102 (53)	64 (52)
Splice site	56 (15)	42 (22)	23 (18)
Nonsense	25 (7)	22 (11)	4 (3)
CNV	13 (4)	7 (4)	0
Small indels	47 (13)	20 (10)	34 (27)

CNV, copy number variation; eGFR, estimated glomerular filtration rate; indels, insertion-deletion.

Values for continuous variables are expressed as median (interquartile range); values for categorical data are given as number (percent).

### Prediction Model Construction

The associations of each baseline clinical and genetic factor with the development of kidney failure are presented in Supplementary Table S1. By sex, male patients had a significantly increased risk of progression to kidney failure than females (HR: 4.65, 95% confidence interval [CI]: 2.66–8.11). Patients with hyperlipemia had significantly worse kidney prognoses than those without (HR: 2.20, 95% CI: 1.55–3.14). Patients were more likely to develop kidney failure earlier if they had baseline proteinuria > 3.5 g/24 h, baseline eGFR < 60 ml/min per 1.73 m^2^, hearing loss or hypertension (Supplementary Figure S1). As for the genetic factors, compared with patients who had a missense variant, patients with pathogenic variants of copy number variation, nonsense, frameshift indels, and spice sites were associated with a higher risk of kidney failure. Moreover, patients harboring truncating *COL4A5* variants were more likely to develop kidney failure earlier than patients with nontruncating variants (HR: 2.59, 95% CI: 1.79–3.76).

**Figure 1:**
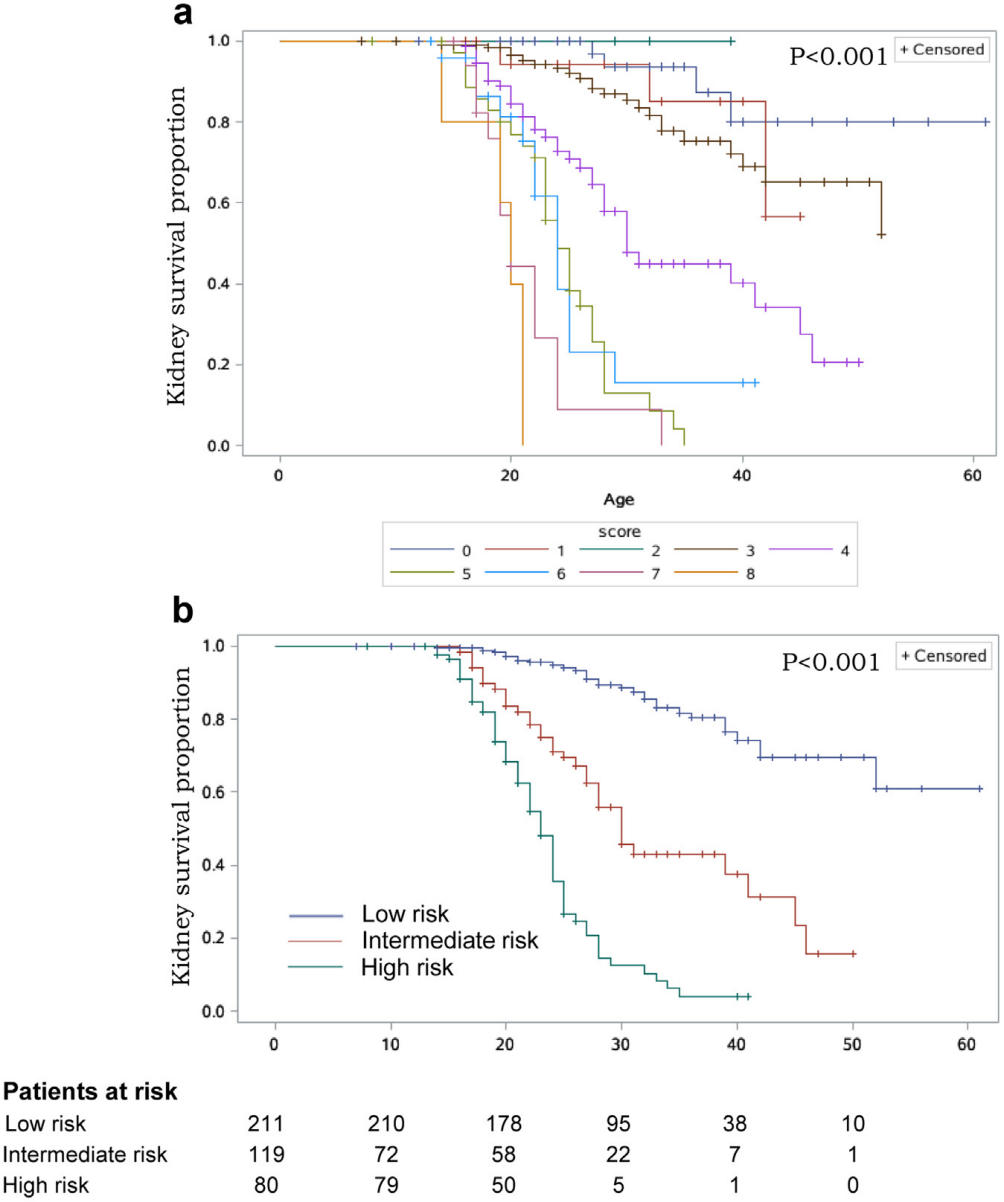
The clinical plus genetic model enables stratification of the risk of progression to kidney failure in patients with XLAS. (a) Kidney survival in patients with the scores ranging from 0 to 8 points. (b) Significant differences in kidney survival in patients with XLAS from the 3 prognostic groups in the development cohort: low risk (0–3 points), intermediate risk (4 points), and high risk (7–8 points). XLAS, X-linked Alport syndrome.

In multivariate analysis with stepwise selection, the following 4 variables remained as independent predictors of the progression to kidney failure among patients with XLAS: sex, baseline proteinuria, baseline eGFR, and the pathogenic variants in *COL4A5* (Table 2).^2^ The results were confirmed by the internal cross-validation (Supplementary Table S2). All variables fulfilled the proportional hazard assumption. In the scoring system based on the clinical plus genetic model, baseline proteinuria > 3.5 g/24 h and baseline eGFR < 60 ml/min per 1.73 m^2^ both accounted for 1 point whereas being a man accounted for 3 points. As for the pathogenic variants in *COL4A5,* there was 0 point for missense variants or in-frame indels, 1 point for splice site, 2 points for frameshift indels, and 3 points for copy number variation or nonsense variants. The total risk score was calculated as the sum of the scores for the 4 variables, ranging from 0 to 8 points. According to the Kaplan-Meier curves of kidney survival by patients with different scores, 3 risk groups could be defined, with patients at low, intermediate, and high risks of the progression to kidney failure scoring 0 to 3, 4, and 5 to 8 points, respectively (Figure 1). The result was confirmed by the cross-validation (Supplementary Figure S2).

**Table 2:** Multivariate Cox proportional hazards regression and the clinical plus genetic model (*n* = 363)

Variables	*n*	HR (95% CI)	*P* value^2^	Score
Sex				
Male	254	5.75 (3.16–10.49)	<0.001	3
Female	109	Ref.		0
Proteinuria, g/24h				
< 3.5	277	Ref.		0
≥ 3.5	86	2.83 (1.87–4.29)	<0.001	1
eGFR, ml/min per 1.73 m^2^				
≥ 60	305	Ref.		0
< 60	58	2.19 (1.48–3.24)	< 0.001	1
Type of pathogenic variants				
Missense	222	Ref.		
Splice site	56	2.35 (1.4–3.96)	0.001	1
Nonsense	25	5.95 (2.86–12.35)	< 0.001	3
CNV	13	6.13 (2.71–13.85)	< 0.001	3
Frameshift indels	38	5.07 (3.02–8.51)	< 0.001	2
In-frame indels	9	2.16 (0.52–8.96)	0.288	0

CI, confidence interval; CNV, copy number variation; eGFR, estimated glomerular filtration rate; HR, hazard ratio; indels, insertion-deletion.

Based on the risk groups, the median kidney survival time of patients at low, intermediate, and high risk were 61 years, 30 (IQR: 24–45) years, and 23 (IQR: 19–25) years, respectively (Table 3, Supplementary Figure S3). Compared with patients with XLAS at low risk, patients at intermediate risk were significantly associated with an increased risk of kidney failure (HR: 4.57, 95% CI: 2.79–7.48), as well as patients at high risk (HR: 14.86, 95% CI: 9.29–23.77). This significant risk stratification was also presented in the subgroup analysis stratified by the baseline characteristics (Supplementary Figure S4).

**Table 3:** Comparisons of prognostic outcomes in patients from the low-, intermediate-, and high-risk groups, based on Kaplan-Meier and Cox regression in the Development cohort

Risk group	Patients, *n/N*	Kaplan-Meier analysis		Cox analysis	
		Median age at kidney failure (IQR) (yr)	Risk of kidney failure at age 30 yr ± SEM (%)	HR (95% CI)	*P* value^a^
Low risk	31/211	61^b^	12 ± 3	1.00	
Intermediate risk	34/72	30 (24–45)	54 ± 7	4.57 (2.79–7.48)	< 0.001
High risk	59/80	23 (19–25)	89 ± 4	14.86 (9.29–23.77)	< 0.001

CI, confidence interval; HR, hazard ratio; IQR, interquartile range.

a The *P* value between intermediate risk and high risk is < 0.001.

b 15% of the patients in the low-risk group developed kidney failure at a median age of 61 years.

As for the outcome of the 5-year and 10-year risk of kidney failure, the risk stratification was significant according to the risk groups (Supplementary Table S3). There was a clear gradient in the risk of kidney failure in patients at low, intermediate, and high risk within 5 years (7%, 31%, and 56%, respectively) and 10 years (12%, 43%, and 74%, respectively) (*P* < 0.05 for all comparisons).

### Prediction Model Performance

In Table 4, we show the performance of the clinical plus genetic model in the prediction of the onset of kidney failure at different ages at the cutoff points 3 and 4, which were the thresholds between risk groups. The performances of the score at other cutoff points are presented in Supplementary Table S4. We can find that at the age of 30 years, the clinical plus genetic model shows the best discrimination to predict kidney survival, with an AUC of 0.83 (95% CI: 0.78–0.88). The score ≤ 3 allows the possibility of kidney failure onset before the age of 30 years to be eliminated with a high negative predictive value of 92%, a sensitivity of 83%, and a specificity of 73%. The probabilities of kidney failure onset before the age of 30 years were 12% in the low-risk group, 54% in the intermediate-risk group, and 89% in the high-risk group (Table 3). According to the subgroup analysis based on the baseline characteristics, the AUCs for the clinical plus genetic model predicting renal survival before the age of 30 years were all > 0.75, showing excellent accuracy and robustness (Supplementary Figure S5). In some subgroups such as XLAS patients without ACEI or ARB treatment, the AUC was high to 0.89 (95%CI: 0.83–0.95). For predicting the outcome that progressed to kidney failure within 5 years and 10 years of follow-up, the clinical plus genetic model presented an excellent performance, with AUCs > 0.81 and well calibration plots (Supplementary Figure S6).

**Table 4:** Performance characteristics of the clinical plus genetic model for the cutoff points (or thresholds) of 3 and 4 (delineating the low-, intermediate-, and high-risk groups) and for different censored ages in the Development cohort

Cutoff scores and censored age	Sensitivity (%)	Specificity (%)	PPV (%)	NPV (%)
3 (inferior or equal to vs. superior)				
20	90 ± 5	63 ± 3	18 ± 3	99 ± 1
25	87 ± 4	69 ± 3	40 ± 3	96 ± 1
30	83 ± 4	73 ± 3	53 ± 4	92 ± 2
35	79 ± 4	75 ± 3	58 ± 4	89 ± 2
40	77 ± 4	75 ± 3	59 ± 4	87 ± 2
45	75 ± 4	75 ± 3	60 ± 4	86 ± 2
4 (inferior or equal to vs. superior)				
20	65 ± 9	81 ± 2	25 ± 5	96 ± 1
25	62 ± 6	88 ± 2	55 ± 6	90 ± 2
30	57 ± 5	91 ± 2	69 ± 5	85 ± 2
35	52 ± 5	91 ± 2	72 ± 5	81 ± 2
40	50 ± 5	91 ± 2	74 ± 5	80 ± 2
45	49 ± 5	91 ± 2	74 ± 5	78 ± 2

NPV, negative predictive value; PPV, positive predictive value.

The test result is positive when the score is greater than the cutoff point and negative when the score is less than or equal to the cutoff point.

**Figure 2:**
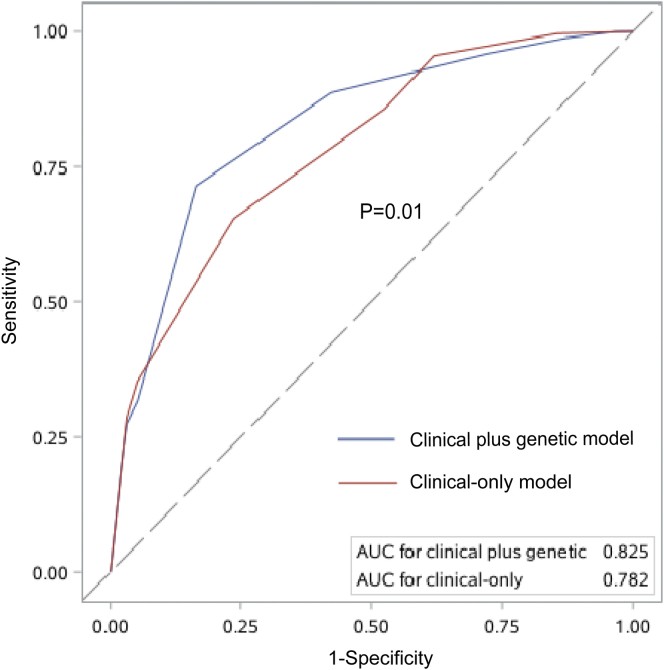
Performance comparison for predicting the onset of kidney failure before age 30 between the clinical plus genetic and clinical-only models in the Development cohort. AUC, area under the curve.

In the clinical-only model, there were 4 variables remained for predicting the onset of kidney failure in XLAS, that is, sex, baseline proteinuria, baseline eGFR, and hearing loss (Supplementary Table S5). Based on this model with a total of 6 points, being a male and baseline proteinuria > 3.5 g/24 h both accounted for 2 points, whereas baseline eGFR < 60 ml/min per 1.73 m^2^ and the presence of hearing loss accounted for 1 point. When comparing the performance for predicting the onset of kidney failure before the age of 30 years, the clinical model also presented a good prediction with an AUC of 0.79 (95% CI: 0.74–0.84); however, it was less accurate than the clinical plus genetic model (*P* = 0.01, Figure 2).

### External Validation

The clinical plus genetic model also showed a similar risk classification and excellent discrimination ability in predicting the progression to kidney failure in 2 external cohorts (Supplementary Table S6 and Figure S7). Based on the risk scoring, the median age for kidney failure of the 3 risk groups was 62 years, 27 (IQR: 19–38) years, and 21 years, respectively; however, the risk of kidney failure between patients at intermediate risk and high risk was not significant (*P* = 0.2). The results were similar in the database cohort.

**Figure 3:**
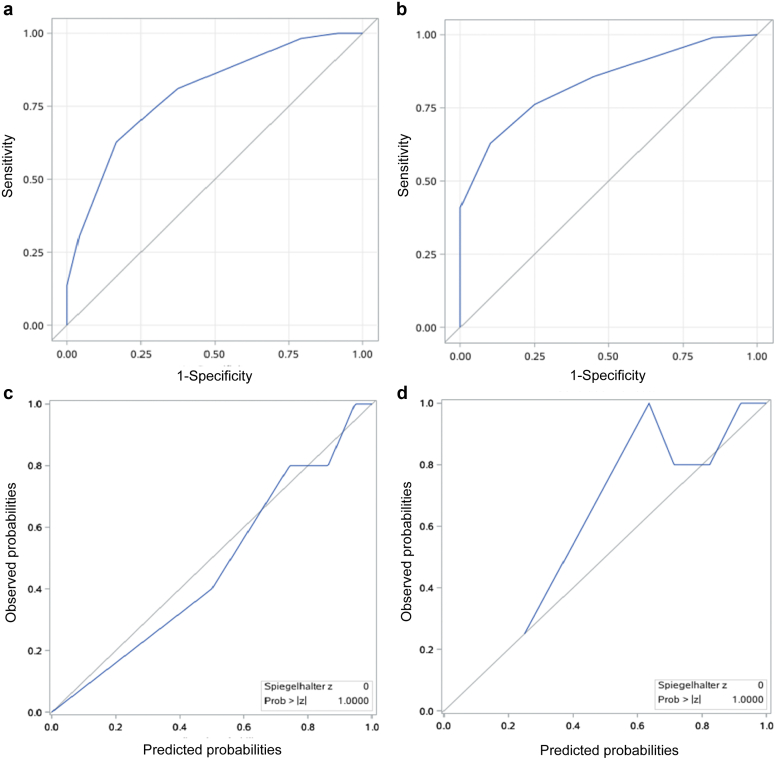
Receiver operating curves and calibration curves for discrimination of the development of kidney failure before age 30 in the external cohorts. (a) Receiver operating curves in the multicenter cohort. (b) Receiver operating curve in the database cohort. (c) Calibration curve in the multicenter cohort. (d) Calibration curve in the database cohort.

For predicting the outcome that progressed to kidney failure before the age of 30 years, the AUC was 0.80 (95% CI: 0.71–0.89) in the multicenter cohort, as well as a smoothed calibration curve (Figure 3). With the cutoff value of 3 points in the risk score, the sensitivity was 83% and the specificity was 63%, with a high negative predictive value of 97% (Supplementary Table S7). In the database cohort, the AUC was high to 0.84 (95% CI: 0.76–0.91), though its calibration plot appeared not very well.

### Stratified Models by Sex

Considering the significant difference in disease progression in male and female patients with XLAS, we tried to build the stratified models by sex, though with insufficient data. In male patients, the model was the same as the original model (Supplementary Table S8 and Figure S8). However, in females, who were all heterozygous in our study, the model was changed and with a total risk score of 3 points: hyperlipemia (2 points) and transcript type (1 point). Two risk groups were defined for the females based on this model with a cutoff score of 3 points, which was similar to the original model, though the predictors were different (Supplementary Table S9, Figure S9, and Figure S4).

## Discussion

As an inherited kidney disease, AS has significant clinical heterogeneity, with patients progressing to kidney failure at ages ranging from < 20 to > 60 years. Previous research has reported the genotype-phenotype correlations in AS. In 2000, a study found among 401 male patients belonging to 195 families with pathogenic variants in *COL4A5* , large deletions, nonsense variants, or small variants changing the reading frame conferred to affected patients a 90% probability of developing kidney failure before the age of 30 years, whereas the same risk was of 50% and 70% in patients with missense or splice site variants, respectively.^3^ The explanation is that the characteristics of pathogenic variants, including the properties of the altered amino acid, the position within the protein and the type of effect on the protein, can change the structure of the *α*3*α*4*α*5(IV) collagen to varying degrees, resulting in different impacts on disease progression.^23^ As a result, genotype-phenotype correlations can be applied to develop prognosis models targeting predicting kidney outcomes and risk stratification. In nephrology, there was a good example of this application in autosomal dominant polycystic kidney disease.^22^ However, there is no similar prognostic tool for AS currently, which could be helpful to personalize clinical decision-making.

To develop a prediction model, the availability of a large data set with detailed genetic and clinical information is important for good performance. Taking advantage of next-generation sequencing technology in the clinic, genetic variants screening has become more available, efficient and cost-effective, enabling the application of genetic data for routine diagnosis or research in AS.^24^^,^^25^ All of the pathogenic variants in *COL4A5* detected in our development cohort are listed in Supplementary Table S10. Because AS is a rare disease with a long course, it is difficult to recruit enough cases with complete information to develop a prediction model for all genetic patterns of AS in our center. As a result, we focused on patients with XLAS and conducted this retrospective cohort study with a long follow-up period, and integrated the genetic variant with clinical data, hoping to build a prognostic model to predict kidney outcomes based on an individual’s baseline information.

The variables that remained in the final model were independently associated with progression to kidney failure, which was consistent with previous studies.^7^^,^^26^ For hearing loss, because some studies have reported its association with the genotypes*,*^23^ its presence might serve as an alternative to the pathogenic variants in *COL4A5* when genetic testing is not available, as in our clinical-only model. However, because the performance of the clinical plus genetic model is significantly better than the clinical-only model, this suggests that genetic testing is necessary and helpful for patients with XLAS in clinical practice to predict the progression to kidney failure.

The accuracy of the clinical plus genetic model in predicting the development of kidney failure before the age of 30 years was excellent in our development cohort, with an AUC of 0.83. The high negative predictive value means that 92% of the patients with XLAS who scored ≤ 3 would not develop kidney failure before the age of 30 years, which could be a useful tool for risk prediction and the provision of appropriate genetic counseling in XLAS. In addition, it is a robust prognostic tool for its significant risk stratification and high AUCs among different subgroups, regardless of the patients with hypertension, ACEI or ARB treatment and other baseline characteristics or not. In addition, the performance of the clinical plus genetic model in 2 external cohorts shows that it has good extensibility, even though the multicenter cohort is much younger than our development cohort. Because of its excellent predictive performance in almost all subgroups, we thought this model, which included all patients with XLAS, might be better than the stratified models by sex when put into the clinical practice.

To our knowledge, this is the first prediction model based on genetic variants and clinical factors developed in XLAS, targeting to predict the progression to kidney failure in clinical care. The strength of our study is the retrospective cohort with a long follow-up, which provided all types of pathogenic variants, detailed clinical information, and adequate events of kidney failure for developing the model. Second, model robustness and extensibility were evaluated by subgroup analysis and external validation, which led to highly consistent findings. However, this study has some limitations. First, the sample size was still relatively small, especially for some subgroups, which limited us to develop more accurate stratified models by baseline age or sex, because there were significant differences in disease progression between these subgroups. Second, because our center primarily accepted patients aged > 16 years, severe cases with an early onset might be missed in our development cohort. Moreover, it was difficult for us to build the models stratified by the baseline age because of the small sample size of children, though there seemed to be a significant difference in the risk of kidney failure between adults and children. However, the impact of this selection bias might be limited because the prediction model showed excellent performance in the external cohorts that included younger cases. Third, because the model was developed and validated in Chinese cohorts, whether it can apply to other regions or all ethnic groups remains to be established.

In our study, a prediction model of progression to kidney failure based on clinical characteristics and genetic variants was developed and validated in patients with XLAS. We argue that this model will be valuable and applicable to clinical practice in assisting with disease risk assessment and management in XLAS.

## Disclosure

All the authors declared no competing interests.

## Supplementary Material

Supplementary File (PDF) **Figure S1.** Probability of progression to kidney failure based on baseline characteristics of patients with XLAS in the development cohort. (A) Sex, (B) proteinuria, (C) hearing loss, (D) eGFR, (E) pathogenic variants in COL4A5, and (F) hypertension. **Figure S2.** Comparison of kidney survival curves in the 3risk groups, low (blue), intermediate (red), and high (green) risk of progression to kidney failure, in the developmental cohort (solid curves) and the 10 training data set pooled together (dotted curves). Kidney survival in the developmental cohort and the 10 training data set pooled together did not differ. **Figure S3.** The scoring system of the clinical plus genetic model helps to predict the disease progression of XLAS. **Figure S4.** Subgroup analysis of prognostic outcomes comparisons in patients from the low-, intermediate-, and high-risk groups, based on Kaplan-Meier and Cox proportional hazards regression, in the development cohort. **Figure S5.** Subgroup analysis of AUC for the clinical plus genetic model predicting the onset of kidney failure before 30 years of age in the development cohort. **Figure S6.** Receiver operating curves (ROC) and calibration curves for discrimination of the onset of kidney failure in the Development cohort. (A) ROC of the 5-year risk of kidney failure (AUC = 0.812), (B) ROC of the 10-year risk of kidney failure (AUC = 0.817), (C) calibration curve for the 5-year risk of kidney failure, and (D) Calibration curve for the 10-year risk of kidney failure. **Figure S7.** Probability of progression to kidney failure based on the risk groups in the external cohorts. (A) the multicenter cohort and (B) the database cohort. **Figure S8.** The clinical plus genetic model enables stratification of the risk of progression to kidney failure in males with XLAS. (A) Kidney survival in patients with the scores ranging from 0 to 5 points, (B) significant differences in kidney survival in XLAS males from the 3risk groups, as follows: low risk (0 point), intermediate risk (1 point), and high risk (2–5 points). **Figure S9.** The clinical plus genetic model enables stratification of the risk of progression to kidney failure in females with XLAS. (A) Kidney survival in patients with the scores ranging from 0 to 3 points, (B) significant differences in kidney survival in XLAS females from the 2risk groups: low risk (0–2 points) and high risk (3 points). **Table S1.** Baseline characteristics and univariate Cox proportional hazards regression (*n* = 363). **Table S2.** The predictive value of the data-derived scores was evaluated using 10-fold cross-validation. **Table S3.** Comparisons of other prognostic outcomes in patients from the low-, intermediate-, and high-risk groups, based on logistic regression in the development cohort. **Table S4.** Performance characteristics of the clinical plus genetic model for the additional cut-off points (or thresholds) of 1, 2, 5, 6, and 7 (in complement to the main data presented in Table 4) and for different censored ages in the development cohort. **Table S5.** Multivariate Cox proportional hazards regression for the clinical-only model (*n* = 313). **Table S6.** Prognostic outcomes in patients from external cohorts stratified by risk groups, based on Kaplan-Meier and Cox regression. **Table S7.** Performance characteristics of the clinical plus genetic model for the cutoff points (or thresholds) of 3 and 4 (delineating the low-, intermediate-, and high-risk groups) and for different censored ages in the 2external cohorts. **Table S8.** Multivariate Cox proportional hazards regression and the clinical plus genetic model for male XLAS (*n* = 254). **Table S9.** Multivariate Cox proportional hazards regression and the clinical plus genetic model for female XLAS (*n* = 97). **Table S10.** Detailed information of pathogenic variants in COL4A5 of 363 patients with XLAS. STROBE Checklist.

## Footnotes

Supplementary File (PDF)
**Figure S1.** Probability of progression to kidney failure based on baseline characteristics of patients with XLAS in the development cohort. (A) Sex, (B) proteinuria, (C) hearing loss, (D) eGFR, (E) pathogenic variants in COL4A5, and (F) hypertension. **Figure S2.** Comparison of kidney survival curves in the 3 risk groups, low (blue), intermediate (red), and high (green) risk of progression to kidney failure, in the developmental cohort (solid curves) and the 10 training data set pooled together (dotted curves). Kidney survival in the developmental cohort and the 10 training data set pooled together did not differ. **Figure S3.** The scoring system of the clinical plus genetic model helps to predict the disease progression of XLAS. **Figure S4.** Subgroup analysis of prognostic outcomes comparisons in patients from the low-, intermediate-, and high-risk groups, based on Kaplan-Meier and Cox proportional hazards regression, in the development cohort. **Figure S5.** Subgroup analysis of AUC for the clinical plus genetic model predicting the onset of kidney failure before 30 years of age in the development cohort. **Figure S6.** Receiver operating curves (ROC) and calibration curves for discrimination of the onset of kidney failure in the Development cohort. (A) ROC of the 5-year risk of kidney failure (AUC = 0.812), (B) ROC of the 10-year risk of kidney failure (AUC = 0.817), (C) calibration curve for the 5-year risk of kidney failure, and (D) Calibration curve for the 10-year risk of kidney failure. **Figure S7.** Probability of progression to kidney failure based on the risk groups in the external cohorts. (A) the multicenter cohort and (B) the database cohort. **Figure S8.** The clinical plus genetic model enables stratification of the risk of progression to kidney failure in males with XLAS. (A) Kidney survival in patients with the scores ranging from 0 to 5 points, (B) significant differences in kidney survival in XLAS males from the 3 risk groups, as follows: low risk (0 point), intermediate risk (1 point), and high risk (2–5 points). **Figure S9.** The clinical plus genetic model enables stratification of the risk of progression to kidney failure in females with XLAS. (A) Kidney survival in patients with the scores ranging from 0 to 3 points, (B) significant differences in kidney survival in XLAS females from the 2 risk groups: low risk (0–2 points) and high risk (3 points). **Table S1.** Baseline characteristics and univariate Cox proportional hazards regression (*n* = 363). **Table S2.** The predictive value of the data-derived scores was evaluated using 10-fold cross-validation. **Table S3.** Comparisons of other prognostic outcomes in patients from the low-, intermediate-, and high-risk groups, based on logistic regression in the development cohort. **Table S4.** Performance characteristics of the clinical plus genetic model for the additional cut-off points (or thresholds) of 1, 2, 5, 6, and 7 (in complement to the main data presented in Table 4) and for different censored ages in the development cohort. **Table S5.** Multivariate Cox proportional hazards regression for the clinical-only model (*n* = 313). **Table S6.** Prognostic outcomes in patients from external cohorts stratified by risk groups, based on Kaplan-Meier and Cox regression. **Table S7.** Performance characteristics of the clinical plus genetic model for the cutoff points (or thresholds) of 3 and 4 (delineating the low-, intermediate-, and high-risk groups) and for different censored ages in the 2 external cohorts. **Table S8.** Multivariate Cox proportional hazards regression and the clinical plus genetic model for male XLAS (*n* = 254). **Table S9.** Multivariate Cox proportional hazards regression and the clinical plus genetic model for female XLAS (*n* = 97). **Table S10.** Detailed information of pathogenic variants in COL4A5 of 363 patients with XLAS. STROBE Checklist.
