# Supplementary material for: A Prediction Model of Disease Progression in X-Linked Alport syndrome Based on Clinical Characteristics and Genetic Variants
Source: Kidney Int Rep. 2025 Mar 10;10(6):2024–34. doi: 10.1016/j.ekir.2025.03.006 (PMC12231008; doi:10.1016/j.ekir.2025.03.006)
Supplement: Supplementary File (PDF) — Figure S1. Probability of progression to kidney failure based on baseline characteristics of patients with XLAS in the development cohort. (A) Sex, (B) proteinuria, (C) hearing loss, (D) eGFR, (E) pathogenic variants in COL4A5, and (F) hypertension. Figure S2. Comparison of kidney survival curves in the 3risk groups, low (blue), intermediate (red), and high (green) risk of progression to kidney failure, in the developmental cohort (solid curves) and the 10 training data set pooled together (dotted curves). Kidney survival in the developmental cohort and the 10 training data set pooled together did not differ. Figure S3. The scoring system of the clinical plus genetic model helps to predict the disease progression of XLAS. Figure S4. Subgroup analysis of prognostic outcomes comparisons in patients from the low-, intermediate-, and high-risk groups, based on Kaplan-Meier and Cox proportional hazards regression, in the development cohort. Figure S5. Subgroup analysis of AUC for the clinical plus genetic model predicting the onset of kidney failure before 30 years of age in the development cohort. Figure S6. Receiver operating curves (ROC) and calibration curves for discrimination of the onset of kidney failure in the Development cohort. (A) ROC of the 5-year risk of kidney failure (AUC = 0.812), (B) ROC of the 10-year risk of kidney failure (AUC = 0.817), (C) calibration curve for the 5-year risk of kidney failure, and (D) Calibration curve for the 10-year risk of kidney failure. Figure S7. Probability of progression to kidney failure based on the risk groups in the external cohorts. (A) the multicenter cohort and (B) the database cohort. Figure S8. The clinical plus genetic model enables stratification of the risk of progression to kidney failure in males with XLAS. (A) Kidney survival in patients with the scores ranging from 0 to 5 points, (B) significant differences in kidney survival in XLAS males from the 3risk groups, as follows: low risk (0 point), intermediate [file mmc1.pdf]

Figure S1. Probability of progression to kidney failure based on baseline characteristics of patients with XLAS in the Development cohort. (A) sex. (B) proteinuria. (C) hearing loss. (D) eGFR. (E) pathogenic variants in COL4A5. (6) hypertension

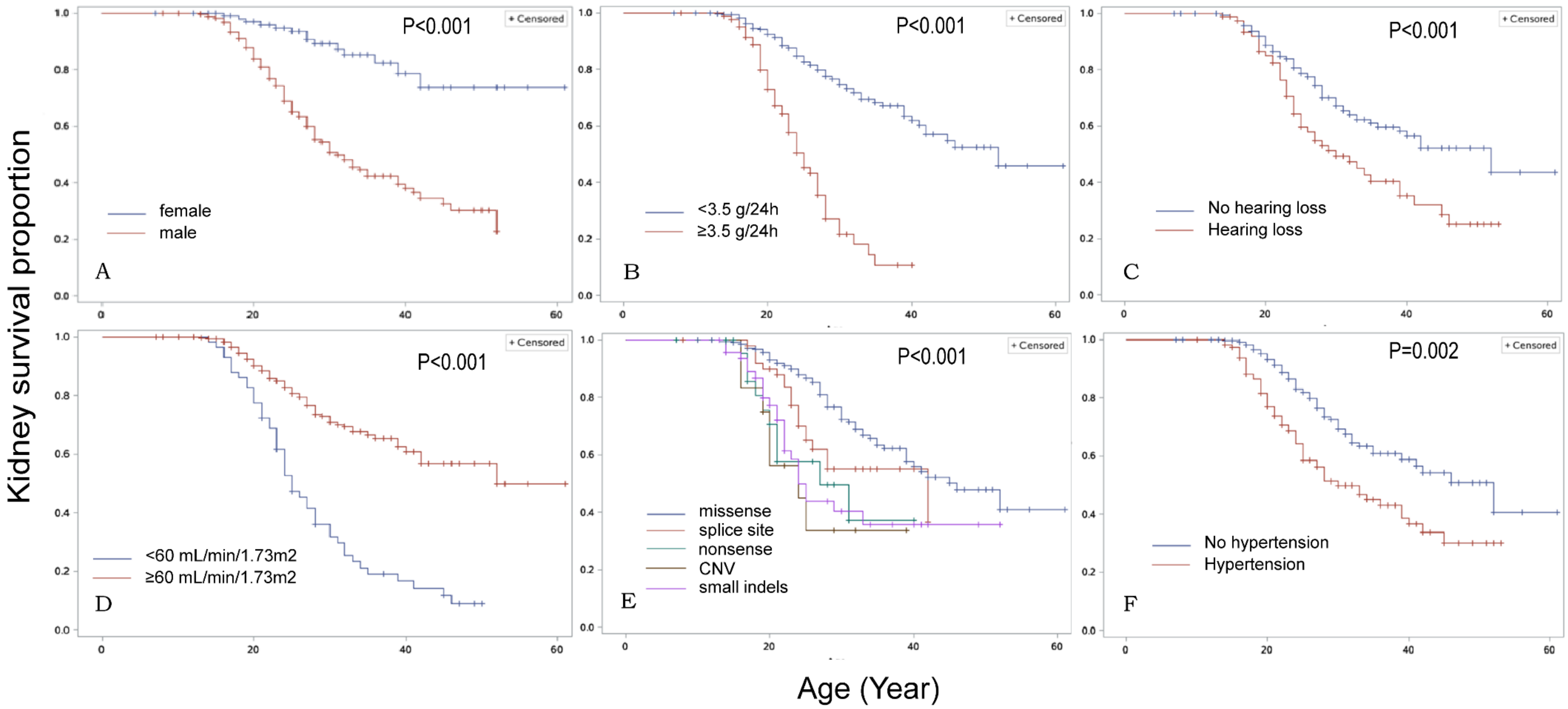

Figure S2. Comparison of kidney survival curves in the three risk groups, low (blue), intermediate (red) and high (green) risk of progression to kidney failure, in the developmental cohort (solid curves) and the 10 training data-set pooled together (dotted curves). Kidney survival in the developmental cohort and the 10 training data-set pooled together did not differ.

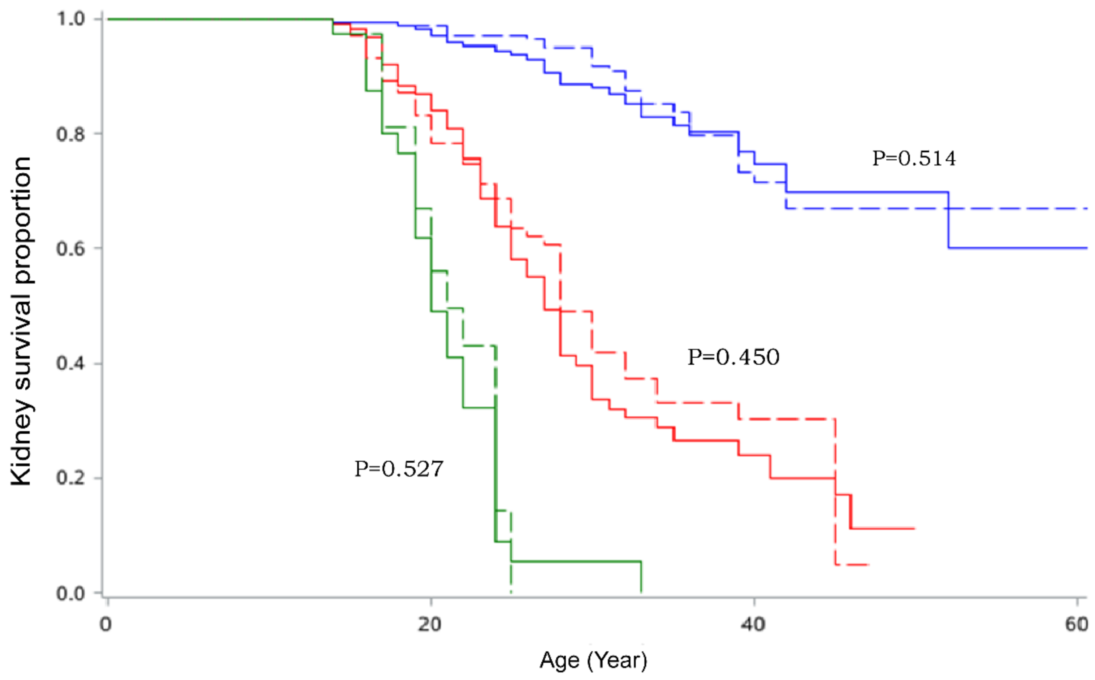

Figure S3 The Scoring system of the clinical plus genetic model helps to predict the disease progression of XLAS

Variable	Points
**Being male**	**3**
**Baseline proteinuria ≥3.5 g/24h**	**1**
**Baseline eGFR <60 mL/min/1.73m^2**	**1**
**Types of Pathogenic variants in *COL4A5***	
**missense/in-frame indels**	**0**
**splice site**	**1**
**frameshift indels**	**2**
**CNV/nonsense**	**3**
**SCORE = Sum**	

SCORE	0	1	2	3	4	5	6	7	8
Risk Assessment	LOW<br>61 median age for kidney failure<br>12% progression to kidney failure at Age 30				INTERMEDIATE<br>30 median age for kidney failure<br>54% progression to kidney failure at Age 30	HIGH<br>23 median age for kidney failure<br>89% progression to kidney failure at Age 30			

Figure S4. Subgroup analysis of prognostic outcomes comparisons in patients with XLAS from the low-, intermediate-, and high-risk groups, based on Kaplan-Meier and Cox proportional hazards regression, in the Development cohort

Prognostic Group	Patients (n)	Kaplan-Meier Analysis: Median Age at kidney failure (IQR) (yr)	Kaplan-Meier Analysis: Risk of kidney failure at Age 30 yr± SEM (%)	Cox Analysis: HR (95% CI)	Cox Analysis: *P* value
**Children & Adolescent**					
Low risk	57	27	64.39±18.36	1	
Intermediate risk	23	20 (18-27)	90.5±8.83	2.91 (1.28-6.59)	<0.001
High risk	37	19 (17-21)	100	5.99 (2.80-12.79)	<0.001
**Adult**					
Low risk	154	61	5.91±2.18	1	
Intermediate risk	49	39 (28-46)	41.77±8.57	4.82 (2.57-9.05)	<0.001
High risk	43	25 (23-28)	81.57±6.60	17.91 (9.94-32.27)	<0.001
**Male**					
Low risk	105	52	17.05±4.61	1	
Intermediate risk	69	30 (24-45)	51.45±7.52	3.26 (1.82-5.84)	<0.001
High risk	80	23 (19-26)	89.10±4.39	10.06 (5.80-17.47)	<0.001
**Female**					
Low risk	106	61	7.49±2.98	1	
Intermediate risk	3	23 (16-27)	100	30.15 (7.13-127.52)	<0.001
High risk	0	/	/	/	/
**ACEI/ARB (-)**					
Low risk	61	61	10.65±4.57	1	
Intermediate risk	15	24	72.22±12.59	7.34 (2.74-19.66)	<0.001
High risk	35	21 (17-24)	96.09±3.82	17.24 (7.15-41.56)	<0.001
**ACEI/ARB (+)**					
Low risk	150	61	11.57±3.19	1	
Intermediate risk	57	31 (27-45)	48.81±8.54	4.10 (2.29-7.34)	<0.001
High risk	45	24 (22-28)	81.54±7.14	11.13 (6.29-19.68)	<0.001
**GBM split<50%**					
Low risk	132	61	8.39±2.90	1	
Intermediate risk	26	45 (27-46)	40.23±10.70	4.43 (2.07-9.48)	<.0001
High risk	5	24 (20-24)	100	21.74 (4.44-106.47)	<.0001
**GBM split>=50%**					
Low risk	39	43	29.25±8.93	1	
Intermediate risk	32	28 (22-39)	64.54±10.96	2.46 (1.13-5.39)	0.024
High risk	53	23 (19-26)	84.76±5.90	4.87 (2.42-9.82)	<.0001
**Normal α5 staining of GBM**					
Low risk	98	56	6.63±2.89	1	
Intermediate risk	17	27 (24-31)	69.12±13.62	10.00 (4.36-22.92)	<0.001
High risk	5	25 (24-26)	100	27.16 (6.54-112.87)	<0.001
**Abnormal α5 staining of GBM**					
Low risk	72	52	21.80±6.27	1	
Intermediate risk	36	39 (23-46)	49.92±10.10	2.91 (1.41-6.04)	0.004
High risk	61	22 (19-25)	87.80±5.31	9.62 (5.00-18.53)	<0.001

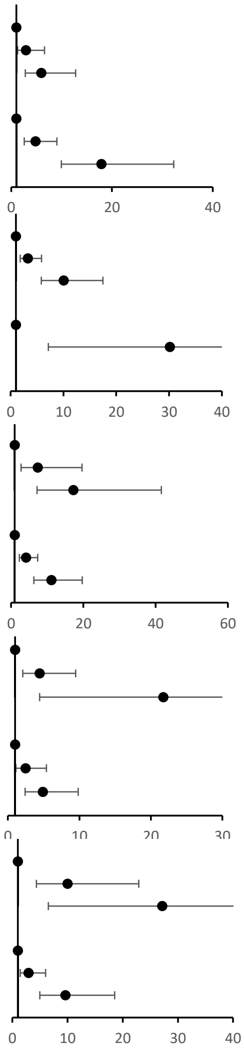

Figure S5 Subgroup analysis of AUC for the clinical plus genetic model predicting the development of kidney failure before 30 years of age in the Development cohort

Subgroup		Patients (n)	AUC (95% CI)
**Overall**		363	0.83 (0.78-0.88)
**Age at diagnosis, years**			
	<18	117	0.76 (0.68-0.85)
	≥18	246	0.88 (0.82-0.93)
**BMI, kg/m^2**			
	<18.5	73	0.83 (0.75-0.92)
	18.5-23.9	174	0.84 (0.76-0.92)
	≥24.0	59	0.88 (0.78-0.99)
**Hyperlipemia**			
	No	236	0.87 (0.81-0.92)
	Yes	127	0.77 (0.68-0.85)
**Hyperuricemia**			
	No	198	0.88 (0.82-0.93)
	Yes	165	0.79 (0.71-0.86)
**Hypertension**			
	No	284	0.82 (0.77-0.88)
	Yes	78	0.83 (0.74-0.92)
**Treatment**			
	ACEI/ARB (-)	111	0.89 (0.83-0.95)
	ACEI/ARB (+)	252	0.80 (0.73-0.86)
**GBM splitting**			
	<50%	163	0.77 (0.65-0.90)
	≥50%	124	0.75 (0.66-0.83)
**α5 staining of GBM**			
	Normal	120	0.87 (0.77-0.96)
	Abnormal	169	0.80 (0.74-0.87)
**Hearing loss**			
	No	131	0.82 (0.73-0.90)
	Yes	116	0.82 (0.75-0.90)
**Ophthalmic examination**			
	Normal	222	0.86 (0.81-0.91)
	Abnormal	34	0.78 (0.63-0.94)
**Transcript type**			
	Non-truncating	251	0.80 (0.73-0.87)
	Truncating	91	0.79 (0.70-0.88)

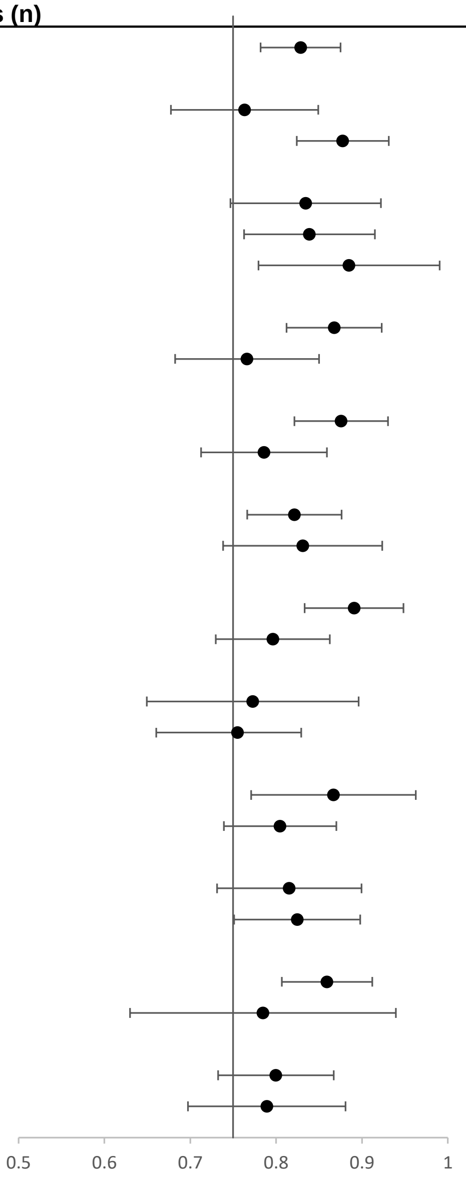

Figure S6. Receiver operating curves (ROC) and calibration curves for discrimination of the onset of kidney failure in the Development cohort. (A) ROC of the 5-year risk of kidney failure (AUC=0.812). (B) ROC of the 10-year risk of kidney failure (AUC=0.817). (C) Calibration curve for the 5-year risk of kidney failure. (D) Calibration curve for the 10-year risk of kidney failure

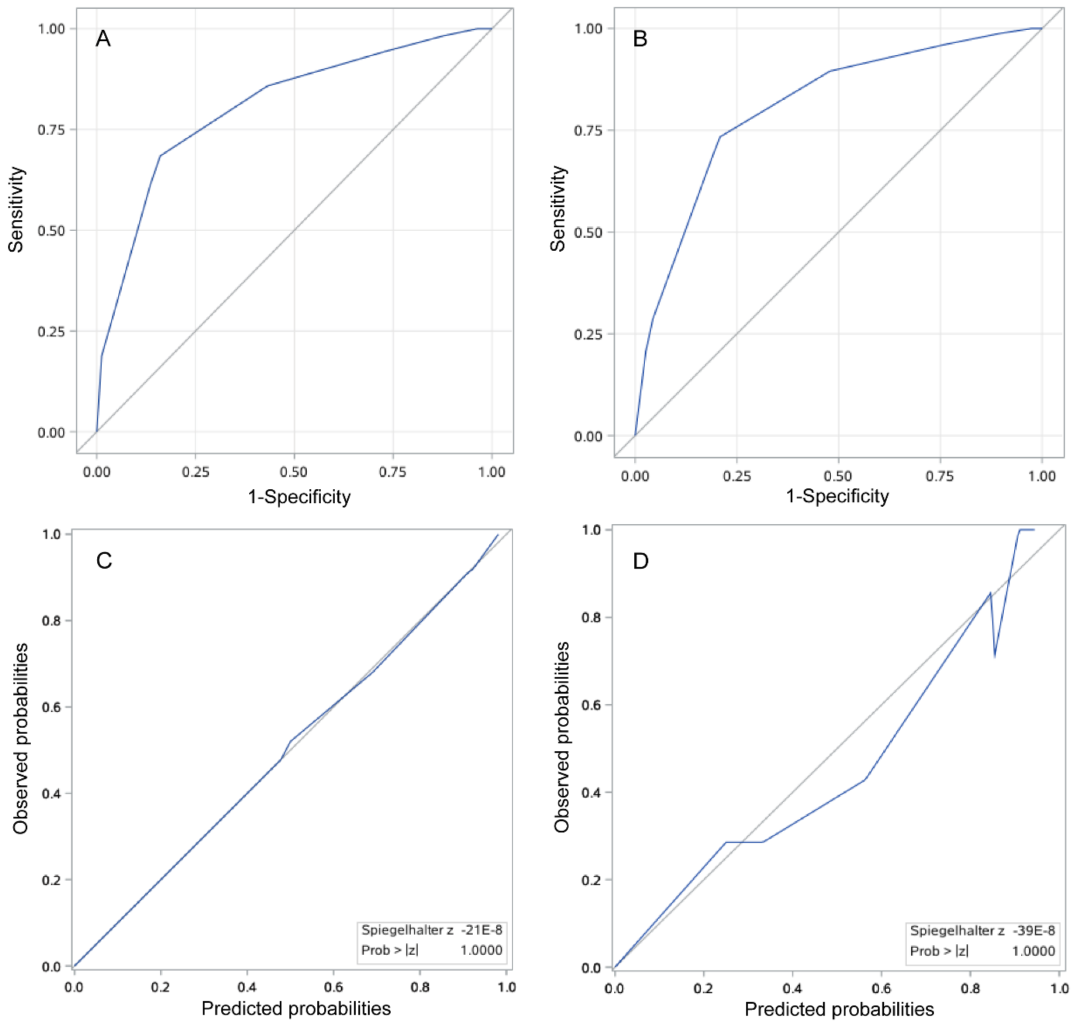

Figure S7. Probability of progression to kidney failure based on the risk groups in the external cohorts. (A) the Multicenter cohort. (B) the Database cohort

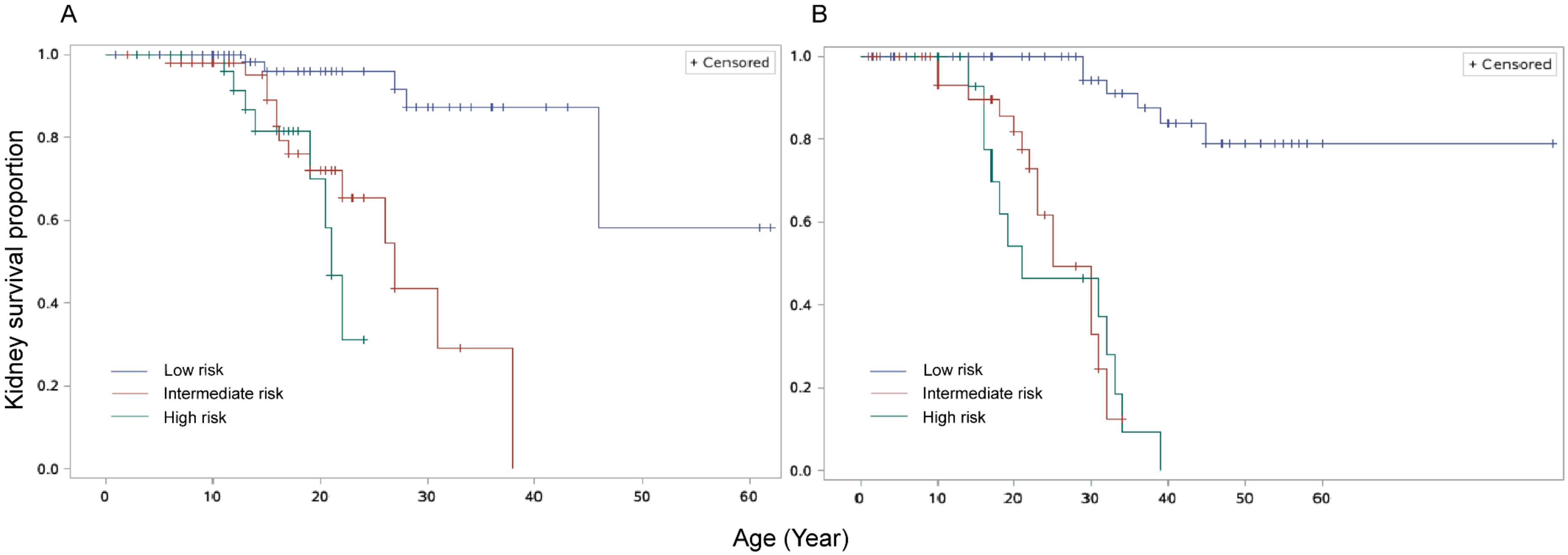

Figure S8 The clinical plus genetic model enables stratification of the risk of progression to kidney failure in males with XLAS. (A) Kidney survival in patients with the scores, ranging from 0 to 5 points. (B) Significant differences in kidney survival in XLAS males from the three risk groups, as follows: low risk (0 point), intermediate risk (1 point), and high risk (2–5 points)

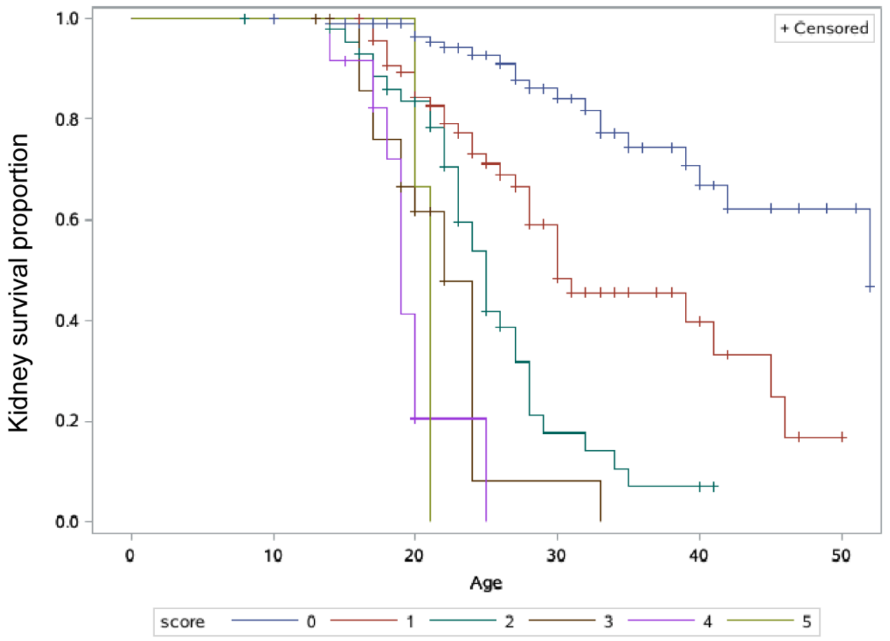

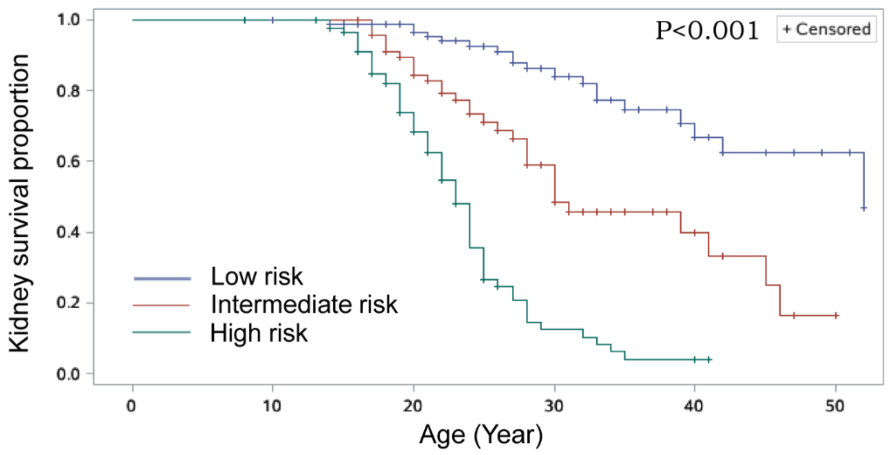

Patients at risk

Low risk	105	105	87	44	19	6
Intermediate risk	69	69	56	22	7	1
High risk	80	79	50	5	1	0

Figure S9 The clinical plus genetic model enables stratification of the risk of progression to kidney failure in females with XLAS. (A) Kidney survival in patients with the scores, ranging from 0 to 3 points. (B) Significant differences in kidney survival in XLAS females from the two risk groups: low risk (0-2 points) and high risk (3 points)

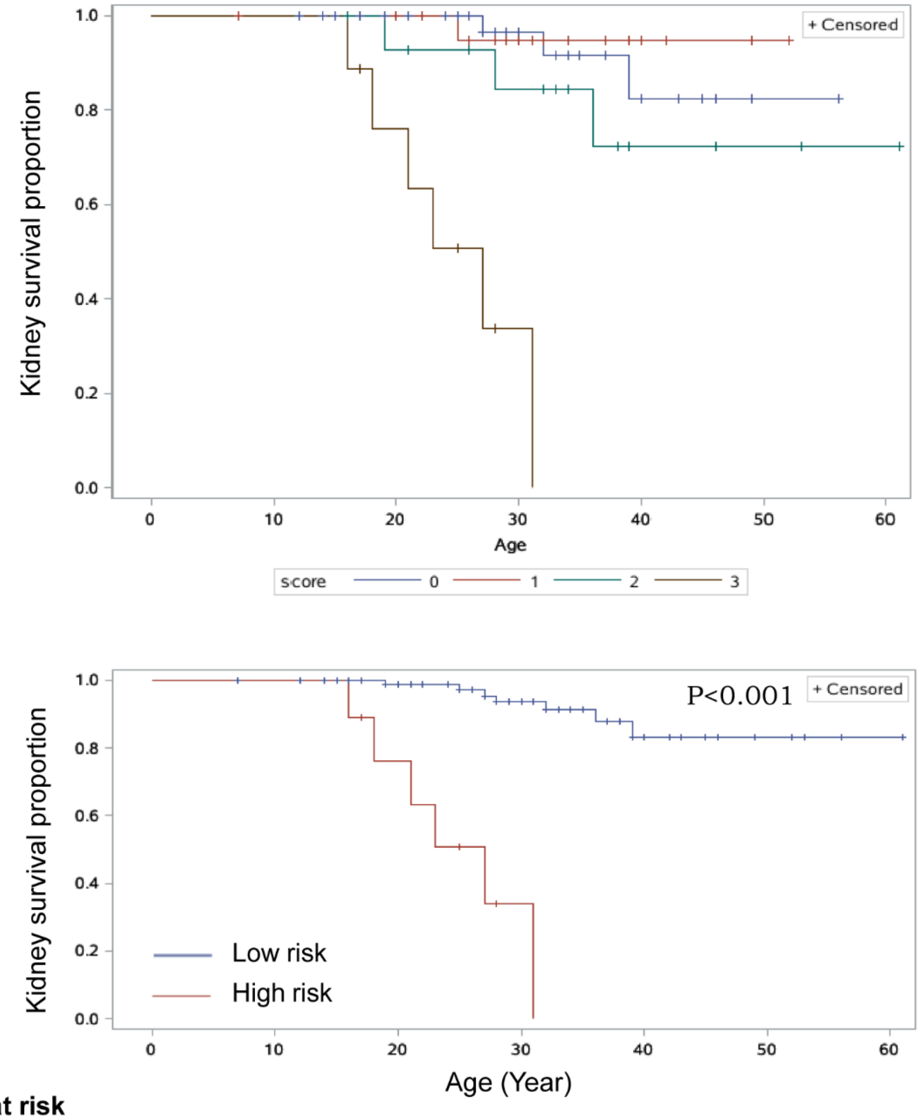

Patients at risk

Low risk	88	87	77	45	17	4
High risk	9	9	6	1	0	

Table S1. Baseline characteristics and univariate Cox proportional hazards regression (N=363)

Characteristics		n (%)	Univariate HR (95%CI)	*P* value
Sex				
	Male	254 (69.97)	4.65 (2.66-8.11)	<0.001
	Female	109 (30.03)	Ref.	
BMI, kg/m^2				
	<18.5	73 (26.16)	0.31 (0.19-0.49)	<0.001
	18.5-23.9	147 (52.69)	Ref.	
	≥24.0	59 (21.15)	0.39 (0.22-0.67)	0.001
	Missing	84		
Glucose, mmol/L				
	<3.9	8 (2.20)	0.25 (0.04-1.82)	0.2
	3.9-6.0	341 (93.94)	Ref.	
	≥6.1	14 (3.86)	0.51 (0.19-1.40)	0.2
Hypertension				
	No	284 (78.45)	Ref.	
	Yes	78 (21.55)	1.82 (1.25-2.63)	0.002
	Missing	1		
Hyperuricemia				
	No	198 (54.55)	Ref.	
	Yes	165 (45.45)	1.31 (0.92-1.86)	0.1
Hyperlipemia				
	No	236 (65.01)	ref	
	Yes	127 (34.99)	2.20 (1.55-3.14)	<0.001
Proteinuria, g/24h				
	<0.4	30 (8.26)	Ref.	
	0.4-3.4	247 (68.04)	5.57 (1.36-22.73)	0.02
	≥3.5	86 (23.69)	22.25 (5.37-92.25)	<0.001
eGFR, mL/min/1.73m^2				
	≥60	305 (84.02)	Ref.	
	<60	58 (15.98)	3.43 (2.39-4.93)	<0.001
Hearing loss				
	No	189 (60.38)	Ref.	
	Yes	124 (39.62)	1.89 (1.31-2.74)	0.001
	Missing	50		
Visual impairment				
	No	222 (86.72)	Ref.	
	Yes	34 (13.28)	0.95 (0.56-1.60)	0.8
	Missing	107		
Smoke				
	No	290 (92.06)	Ref.	
	Yes	25 (7.94)	0.67 (0.34-1.32)	0.2
	Missing	48		
Alcohol				

No	295 (93.95)	Ref.	
Yes	19 (6.05)	0.40 (0.16-0.98)	0.05
Missing	49		
Type of pathogenic variants			
Missense	222 (61.16)	Ref.	
Splice site	56 (15.43)	1.65 (0.99-2.74)	0.05
Nonsense	25 (6.89)	2.86 (1.46-5.60)	0.002
CNV	13 (3.58)	3.50 (1.59-7.67)	0.002
Frameshift indels	38 (10.47)	2.72 (1.67-4.43)	<0.001
In-frame indels	9 (2.48)	1.39 (0.34-5.70)	0.646
Transcript type			
Non-truncating	251 (73.39)	Ref.	
Truncating	91 (26.61)	2.59 (1.79-3.76)	<0.001
Missing	21		

Abbreviation: BMI, body mass index; eGFR, estimated glomerular filtration rate; CNV, copy number variation; indels, insertion-deletion

Table S2 The predictive value of the data-derived scores was evaluated using 10-fold cross-validation.

		C1			C2			C3			C4			C5		
**Variable**	**Category**	**N**	**HR (95%CI)**	**Score**	**N**	**HR (95%CI)**	**Score**	**N**	**HR (95%CI)**	**Score**	**N**	**HR (95%CI)**	**Score**	**N**	**HR (95%CI)**	**Score**
**Sex**	Male	226	4.99 (2.65-9.37)	2	228	6.39 (3.36-12.15)	3	228	6.80 (3.55-13.02)	3	230	6.36 (3.78-12.34)	3	229	6.44 (3.38-12.30)	3
	Female	101	Ref	0	99	Ref	0	99	Ref	0	97	Ref	0	98	Ref	0
**Proteinuria, g/24h**	<3.5	250	Ref	0	248	Ref	0	252	Ref	0	246	Ref	0	251	Ref	0
	≥3.5	77	2.89 (1.86-4.49)	1	79	3.45 (2.22-5.37)	1	75	2.64 (1.70-4.10)	1	81	2.80 (1.81-4.32)	1	76	2.93 (1.89-4.53)	1
**eGFR, mL/min/1.73m^2**	≥60	277	Ref	0	272	Ref	0	280	Ref	0	273	Ref	0	275	Ref	0
	<60	50	2.25 (1.48-3.42)	1	55	1.96 (1.15-2.71)	1	47	2.15 (1.41-3.28)	1	54	2.06 (1.37-3.09)	1	52	2.07 (1.37-3.13)	1
**Type of pathogenic variants**	Missense	200	Ref	0	200	Ref	0	197	Ref	0	297	Ref	0	200	Ref	0
	Splice site	50	2.47 (1.44-4.24)	1	48	2.56 (1.48-4.43)	1	52	2.65 (1.54-4.58)	1	52	2.25 (1.30-3.88)	1	50	2.23 (1.27-3.92)	1
	Nonsense	24	4.91 (2.29-10.53)	2	22	5.70 (2.63-12.35)	3	22	6.90 (3.06-15.58)	3	23	5.38 (2.50-11.56)	3	23	6.08 (2.81-13.19)	3
	CNV	13	5.75 (2.54-13.05)	3	13	6.70 (2.75-16.31)	3	12	6.54 (2.28-18.75)	3	12	7.48 (3.09-18.11)	4	12	7.34 (3.43-18.28)	3
	Frameshift indels	33	5.31 (3.09-9.14)	2	44	5.88 (3.46-9.99)	3	36	5.26 (3.07-9.01)	2	36	4.84 (2.83-8.3)	2	35	5.28 (3.09-9.03)	2
	In-frame indels	7	2.49 (0.60-10.39)	0	0	2.14 (0.51-8.91)	0	8	4.33 (1.03-18.19)	0	7	2.05 (0.49-8.51)	0	7	2.20 (0.16-8.73)	0

Abbreviation: eGFR, estimated glomerular filtration rate; CNV, copy number variation; indels, insertion-deletion.

Table S2 The predictive value of the data-derived scores was evaluated using 10-fold cross-validation (continued)

		C6			C7			C8			C9			C10		
**Variable**	**Category**	**N**	**HR (95%CI)**	**Score**	**N**	**HR (95%CI)**	**Score**	**N**	**HR (95%CI)**	**Score**	**N**	**HR (95%CI)**	**Score**	**N**	**HR (95%CI)**	**Score**
**Sex**	Male	233	6.31 (3.24-12.3)	3	235	6.46 (3.23-12.93)	3	229	5.48 (2.95-10.17)	3	229	5.26 (2.88-9.62)	2	227	6.30 (3.37-11.8)	3
	Female	94	Ref	0	92	Ref	0	98	Ref	0	98	Ref	0	100	Ref	0
**Proteinuria, g/24h**	<3.5	252	Ref	0	252	Ref	0	248	Ref	0	248	Ref	0	252	Ref	0
	≥3.5	75	2.84 (1.83-4.41)	1	75	2.72 (1.76-4.19)	1	79	2.81 (1.82-4.33)	1	79	2.88 (1.87-4.44)	1	75	2.74 (1.77-4.24)	1
**eGFR, mL/min/1.73 m^2**	≥60	277	Ref	0	275	Ref	0	274	Ref	0	273	Ref	0	276	Ref	0
	<60	50	2.27 (1.50-3.43)	1	52	2.45 (1.62-3.71)	1	53	2.02 (1.34-3.04)	1	54	2.21 (1.47-3.31)	1	51	2.13 (1.42-3.21)	1
**Type of pathogenic variants**	Missense	198	Ref	0	206	Ref	0	200	Ref	0	202	Ref	0	198	Ref	0
	Splice site	51	2.26 (1.31-3.92)	1	51	2.73 (1.59-4.7)	1	49	2.23 (1.28-3.89)	1	49	2.36 (1.36-4.11)	1	49	2.57 (1.48-4.48)	1
	Nonsense	21	4.98 (2.22-11.16)	2	22	6.91 (3.06-15.61)	3	23	5.48 (2.56-11.75)	3	20	6.56 (3.14-13.73)	3	23	6.57 (3.12-13.82)	3
	CNV	12	6.47 (2.83-14.76)	3	9	9.94 (5.24-31.10)	4	12	5.43 (2.26-13.03)	3	12	6.09 (2.68-13.83)	3	13	6.12 (2.69-13.96)	3
	Frameshift indels	37	5.14 (2.99-8.83)	2	30	4.76 (2.72-8.32)	2	35	5.00 (2.95-8.49)	2	35	4.85 (2.82-8.34)	2	35	5.02 (2.91-8.64)	2
	In-frame indels	8	2.23 (0.54-9.28)	0	9	2.19 (0.53-9.11)	0	8	2.02 (0.49-8.38)	0	9	2.22 (0.53-9.23)	0	9	2.12 (0.51-8.80)	0

Abbreviation: eGFR, estimated glomerular filtration rate; CNV, copy number variation; indels, insertion-deletion.

Table S3 Comparisons of other prognostic outcomes in patients from the low-, intermediate-, and high-risk groups, based on Logistic regression in the Development cohort

Risk group	**5-year risk of kidney failure**			**10-year risk of kidney failure**		
	n/N (%)	OR (95% CI)	*P* value[1]	n/N (%)	OR (95% CI)	*P* value[2]
Low risk	14/211 (7)	1.00		25/211 (12)	1.00	
Intermediate risk	22/72 (31)	5.88 (3.00-11.52)	<0.001	31/72 (43)	4.95 (2.91-8.40)	<0.001
High risk	45/80 (56)	19.39 (10.39-36.17)	<0.001	59/80 (74)	17.16 (10.43-28.22)	<0.001

[1] The *P* value between intermediate risk and high risk is 0.001
[2] The *P* value between intermediate risk and high risk is 0.001

Table S4. Performance characteristics of the clinical plus genetic model for the additional cut-off points (or thresholds) of 1, 2, 5, 6 and 7 (in complement to the main data presented in Table 4) and for different censored ages.

Cutoff scores and censored age	Sensitivity (%)	Specificity (%)	PPV (%)	NPV (%)
**1 (inferior or equal to versus superior)**				
20	97±3	23±2	10±2	99±1
25	99±1	26±3	25±3	98±2
30	97±2	28±3	33±3	96±2
35	96±2	29±3	38±3	95±3
40	95±2	29±3	39±3	92±3
45	94±2	29±3	40±3	91±3
**2 (inferior or equal to versus superior)**				
20	94±4	26±2	11±2	98±2
25	97±2	30±3	25±3	98±2
30	95±2	32±3	34±3	94±2
35	95±2	33±3	38±3	93±3
40	93±2	33±3	40±3	91±3
45	93±2	33±3	41±3	90±3
**5 (inferior or equal to versus superior)**				
20	39±9	92±1	32±8	94±1
25	35±6	96±1	68±8	86±2
30	27±5	96±1	70±7	78±2
35	24±4	96±1	73±7	74±2
40	23±4	96±1	73±7	72±2
45	22±4	96±1	73±7	71±3
**6 (inferior or equal to versus superior)**				
20	16±7	97±1	36±13	93±1
25	14±4	99±1	71±12	83±2
30	11±3	99±1	79±10	75±2
35	10±3	99±1	79±10	71±2
40	10±3	99±1	79±10	70±2
45	9±3	99±1	79±10	68±2
**7 (inferior or equal to versus superior)**				
20	0±±0	99±1	0±±0	91±1
25	4±2	100±±0	100±±0	81±2
30	3±2	100±±0	100±±0	74±2
35	3±2	100±±0	100±±0	70±2
40	3±1	100±±0	100±±0	68±2
45	2±1	100±±0	100±±0	67±2

Note: The test result is positive when the score is greater than the cutoff point and negative when the score is less than or equal to the cutoff point
Abbreviation: NPV, negative predictive value; PPV, positive predictive value

Table S5. Multivariate Cox proportional hazards regression for the clinical-only model (n=313)

Variables	n	HR (95%CI)	P value	Score
Sex				
Male	220	3.01 (1.69-5.37)	<0.001	2
Female	93	Ref.		0
Proteinuria, g/24h				
<3.5	236	Ref.		0
≥3.5	77	2.84 (1.88-4.28)	<0.001	2
eGFR, mL/min/1.73m2				
≥60	265	Ref.		0
<60	48	2.03 (1.37-3.02)	<0.001	1
Hearing loss				
No	189	Ref.		0
Yes	124	1.66 (1.16-2.38)	0.023	1

Abbreviation: eGFR, estimated glomerular filtration rate

Table S6. Prognostic outcomes in patients from external cohorts stratified by risk groups, based on Kaplan-Meier and Cox proportional hazards regression

Risk group	Patients n/N	**Kaplan-Meier Analysis**		**Cox Analysis**	
		Median Age at kidney failure (IQR) (yr)	Risk of kidney failure at Age 30 yr±SEM (%)	HR (95% CI)	*P* value*
**Multicenter cohort**					
Low risk	5/110	62[a]	13±6	1.00	
Intermediate risk	14/53	27 (19-38)	56±14	9.00 (2.87-28.16)	<0.001
High risk	8/30	21	69±17	15.89 (4.25-59.45)	<0.001
**Database cohort**					
Low risk	6/68	60[b]	6±4	1.00	
Intermediate risk	15/39	25 (22-31)	51±11	19.30 (6.18-60.30)	<0.001
High risk	12/18	21 (17-33)	63±14	20.06 (6.77-59.43)	<0.001

* The *P* value between intermediate risk and high risk is 0.2 in the Multicenter cohort and 0.9 in the Database cohort

[a] 5% of the patients in the low-risk group developed kidney failure at a median age of 62 years

[b] 9% of the patients in the low-risk group developed kidney failure at a median age of 60 years

Table S7. Performance characteristics of the clinical plus genetic model for the cutoff points (or thresholds) of 3 and 4 (delineating the low-, intermediate-, and high-risk groups) and for different censored ages in the two external cohorts

Cutoff scores and censored age	Sensitivity (%)	Specificity (%)	PPV (%)	NPV (%)
**Multicenter cohort**				
**3 (inferior or equal to versus superior)**				
20	88±8	61±4	17±4	98±1
25	90±7	62±4	22±4	98±1
30	83±8	63±4	24±5	97±2
35	84±7	63±4	25±5	97±2
40	85±7	63±4	27±5	97±2
45	85±7	63±4	27±5	97±2
**4 (inferior or equal to versus superior)**				
20	44±12	79±3	16±6	94±2
25	50±11	80±3	23±6	93±2
30	42±10	80±3	23±6	91±2
35	40±10	80±3	23±6	90±2
40	38±10	80±3	23±6	89±3
45	38±10	80±3	23±6	89±3
**Database cohort**				
**3 (inferior or equal to versus superior)**				
20	100±±0	59±5	18±5	100±±0
25	100±±0	62±5	28±6	100±±0
30	90±7	63±5	32±6	97±2
35	90±6	68±5	46±7	96±2
40	84±6	68±5	47±7	93±3
45	84±6	68±5	47±7	93±3
**4 (inferior or equal to versus superior)**				
20	90±9	73±4	23±7	99±1
25	88±8	76±4	35±8	98±2
30	75±10	76±4	38±8	94±3
35	76±8	81±4	55±8	92±3
40	72±8	82±4	58±8	89±3
45	72±8	82±4	58±8	89±3

The test result is positive when the score is greater than the cutoff point and negative when the score is less than or equal to the cutoff point

Abbreviation: NPV, negative predictive value; PPV, positive predictive value

Table S8. Multivariate Cox proportional hazards regression and the clinical plus genetic model for male XLAS (N=254)

Variables	n	HR (95%CI)	*P* value	Score
Proteinuria, g/24h				
<3.5	178	Ref.		0
≥3.5	76	2.45 (1.56-3.84)	<0.001	1
eGFR, mL/min/1.73m2				
≥60	203	Ref.		0
<60	51	2.02 (1.33-3.06)	0.001	1
Type of pathogenic variants				
Missense	161	Ref.		0
Splice site	40	2.70 (1.57-4.65)	<0.001	1
Nonsense	14	5.16 (2.16-12.34)	<0.001	3
CNV	10	6.11 (2.60-14.37)	<0.001	3
Frameshift indels	22	5.69 (3.24-9.99)	<0.001	2
In-frame indels	7	2.60 (0.62-10.87)	0.190	0

Abbreviation: CNV, copy number variation; eGFR, estimated glomerular filtration rate; indels, insertion-deletion.

Table S9. Multivariate Cox proportional hazards regression and the clinical plus genetic model for female XLAS (N=97)

Variables	n	HR (95%CI)	*P* value	Score
Hyperlipemia				
No	83	Ref.		0
Yes	26	9.87 (2.86-34.02)	<0.001	2
Transcript type				
Non-truncating	64	Ref.		0
Truncating	33	4.28 (1.30-14.12)	0.02	1

Table S10 Detailed information of pathogenic variants in COL4A5 of 363 patients with XLAS

Patient ID	Gender	Gene	Exon/ Intron	Zygosity	Nucleotide Change[a]	Amino Acid Change	Type of pathogenic variants
XL1	Male	COL4A5	1	hemi	c.1A>C	p.Met1?	missense
XL2	Male	COL4A5	1	hemi	c.2T>C	p.Met1?	missense
XL3	Female	COL4A5	1	het	c.3G>A	p.Met1?	missense
XL4	Female	COL4A5	1	het	c.15_16del	p.Val6GlnfsTer33	small del/ins/dup
XL5	Male	COL4A5	1	hemi	c.15_16del	p.Val6GlnfsTer33	small del/ins/dup
XL6	Male	COL4A5	1	hemi	c.60G>A	p.Trp20Ter	nonsense
XL7	Male	COL4A5	1	hemi	c.81+2dup		splicing
XL8	Male	COL4A5	2	hemi	c.94_104del	p.Cys32IlefsTer4	small del/ins/dup
XL9	Female	COL4A5	2	het	c.136del	p.Glu46LysfsTer109	small del/ins/dup
XL10	Male	COL4A5	2	hemi	c.136del	p.Glu46LysfsTer109	small del/ins/dup
XL11	Male	COL4A5	3	hemi	c.142G>T	p.Gly48Ter	nonsense
XL12	Female	COL4A5	4	het	c.262C>T	p.Pro88Ser	missense
XL13	Male	COL4A5	4	hemi	c.262C>T	p.Pro88Ser	missense
XL14	Female	COL4A5	5	het	c.314G>A	p.Gly105Asp	missense
XL15	Male	COL4A5	6	hemi	c.348_349insTCCGG	p.Gly117SerfsTer40	small del/ins/dup
XL16	Male	COL4A5	6	hemi	c.350_351insGT	p.Pro118TyrfsTer38	small del/ins/dup
XL17	Male	COL4A5	6	hemi	c.367G>A	p.Gly123Arg	missense
XL18	Female	COL4A5	6	het	c.372del	p.Cys124Ter	small del/ins/dup
XL19	Male	COL4A5	6	hemi	c.385-1G>A		splicing
XL20	Male	COL4A5	7	hemi	c.386G>T	p.Gly129Val	missense

XL21	Female	COL4A5	7	het	c.403G>A	p.Gly135Ser	missense
XL22	Male	COL4A5	7	hemi	c.412G>A	p.Gly138Ser	missense
XL23	Male	COL4A5	7	hemi	c.431G>A	p.Gly144Asp	missense
XL24	Female	COL4A5	7	het	c.438+1G>A		splicing
XL25	Male	COL4A5	7	hemi	c.438+1G>A		splicing
XL26	Male	COL4A5	9	hemi	c.508A>T	p.Lys170Ter	nonsense
XL27	Male	COL4A5	9	hemi	c.538G>A	p.Gly180Arg	missense
XL28	Female	COL4A5	9	het	c.539G>T	p.Gly180Val	missense
XL29	Male	COL4A5	9	hemi	c.546+1G>A		splicing
XL30	Male	COL4A5	10	hemi	c.583G>T	p.Gly195Cys	missense
XL31	Male	COL4A5	10	hemi	c.609+2T>C		splicing
XL32	Male	COL4A5	11	hemi	c.646-12_646-11del		splicing
XL33	Male	COL4A5	11	hemi	c.646-12_646-11del		splicing
XL34	Female	COL4A5	11	het	c.646-1G>A		splicing
XL35	Male	COL4A5	11	hemi	c.646-1G>C		splicing
XL36	Male	COL4A5	11	hemi	c.646-1G>C		splicing
XL37	Male	COL4A5	12	hemi	c.647G>A	p.Gly216Glu	missense
XL38	Male	COL4A5	12	hemi	c.667C>T	p.Gln223Ter	nonsense
XL39	Male	COL4A5	12	hemi	c.668del	p.Gln223ArgfsTer31	small del/ins/dup
XL40	Male	COL4A5	12	hemi	c.686_687+13del		small del/ins/dup
XL41	Male	COL4A5	12	hemi	c.687+2T>A		splicing
XL42	Male	COL4A5	13	hemi	c.688G>A	p.Gly230Ser	missense
XL43	Male	COL4A5	13	hemi	c.688G>T	p.Gly230Cys	missense

XL44	Male	COL4A5	13	hemi	c.689G>T	p.Gly230Val	missense
XL45	Male	COL4A5	13	hemi	c.697G>A	p.Gly233Ser	missense
XL46	Male	COL4A5	13	hemi	c.697G>A	p.Gly233Ser	missense
XL47	Male	COL4A5	13	hemi	c.698G>A	p.Gly233Asp	missense
XL48	Female	COL4A5	13	het	c.761_762del	p.Glu254ValfsTer11	small del/ins/dup
XL49	Female	COL4A5	13	het	c.781-2A>G		splicing
XL50	Female	COL4A5	14	het	c.788del	p.Pro263LeufsTer83	small del/ins/dup
XL51	Female	COL4A5	14	het	c.796C>T	p.Arg266Ter	nonsense
XL52	Male	COL4A5	14	hemi	c.796C>T		nonsense
XL53	Male	COL4A5	14	hemi	c.796C>T	p.Arg266Ter	nonsense
XL54	Female	COL4A5	14	het	c.800G>A	p.Gly267Glu	missense
XL55	Female	COL4A5	14	het	c.800G>A	p.Gly267Glu	missense
XL56	Male	COL4A5	14	hemi	c.834+2T>G		splicing
XL57	Male	COL4A5	14	hemi	c.834+2T>G		splicing
XL58	Male	COL4A5	14	hemi	c.834+5G>A		splicing
XL59	Male	COL4A5	14	hemi	c.834+5G>A		splicing
XL60	Male	COL4A5	14	hemi	c.834+5G>A		splicing
XL61	Female	COL4A5	14	het	c.835-3C>G		splicing
XL62	Male	COL4A5	15	hemi	c.865G>T	p.Gly289Cys	missense
XL63	Male	COL4A5	15	hemi	c.891+1G>A		splicing
XL64	Female	COL4A5	15	het	c.891+5G>A		splicing
XL65	Male	COL4A5	16	hemi	c.928G>A	p.Gly310Arg	missense
XL66	Female	COL4A5	16	het	c.936+1G>C		splicing

XL67	Male	COL4A5	16	hemi	c.936+2T>C		splicing
XL68	Male	COL4A5	17	hemi	c.951_968dup	p.Asp317_Gly322dup	small del/ins/dup
XL69	Male	COL4A5	17	hemi	c.973G>A	p.Gly325Arg	missense
XL70	Male	COL4A5	17	hemi	c.973G>A	p.Gly325Arg	missense
XL71	Male	COL4A5	17	hemi	c.973G>A	p.Gly325Arg	missense
XL72	Male	COL4A5	17	hemi	c.973G>A	p.Gly325Arg	missense
XL73	Male	COL4A5	17	hemi	c.980_983del	p.Asp327ValfsTer18	small del/ins/dup
XL74	Male	COL4A5	17	hemi	c.980_990+74del		small del/ins/dup
XL75	Male	COL4A5	17	hemi	c.983G>A	p.Gly328Asp	missense
XL76	Male	COL4A5	17	hemi	c.990+1G>C		splicing
XL77	Female	COL4A5	17	het	c.991-14A>G		splicing
XL78	Male	COL4A5	17	hemi	c.991-14A>G		splicing
XL79	Female	COL4A5	18	het	c.1001G>A	p.Gly334Asp	missense
XL80	Female	COL4A5	18	het	c.1001G>A	p.Gly334Asp	missense
XL81	Male	COL4A5	18	hemi	c.1001G>A	p.Gly334Asp	missense
XL82	Female	COL4A5	18	het	c.1028G>A	p.Gly343Glu	missense
XL83	Female	COL4A5	18	het	c.1032+1G>A		splicing
XL84	Male	COL4A5	19	hemi	c.1036A>G	p.Ile346Val	missense
XL85	Male	COL4A5	19	hemi	c.1103G>A	p.Gly368Glu	missense
XL86	Male	COL4A5	19	hemi	c.1112_1114del	p.Gly371del	small del/ins/dup
XL87	Male	COL4A5	19	hemi	c.1112_1114del	p.Gly371del	small del/ins/dup
XL88	Male	COL4A5	19	hemi	c.1117C>T	p.Arg373Ter	nonsense
XL89	Male	COL4A5	19	hemi	c.1121G>T	p.Gly374Val	missense

XL90	Female	COL4A5	19	het	c.1147G>C	p.Gly383Arg	missense
XL91	Female	COL4A5	19	het	c.1156G>A	p.Gly386Arg	missense
XL92	Male	COL4A5	19	hemi	c.1165+1G>A		splicing
XL93	Female	COL4A5	19	het	c.1166-2A>T		splicing
XL94	Male	COL4A5	20	hemi	c.1189G>C	p.Gly397Arg	missense
XL95	Male	COL4A5	20	hemi	c.1190G>A	p.Gly397Asp	missense
XL96	Male	COL4A5	20	hemi	c.1190G>A	p.Gly397Asp	missense
XL97	Male	COL4A5	20	hemi	c.1198G>A	p.Gly400Arg	missense
XL98	Male	COL4A5	20	hemi	c.1276G>A	p.Gly426Arg	missense
XL99	Female	COL4A5	21	het	c.1376dup	p.Gly460ArgfsTer5	small del/ins/dup
XL100	Male	COL4A5	21	hemi	c.1378G>A	p.Gly460Arg	missense
XL101	Male	COL4A5	21	hemi	c.1388G>T	p.Gly463Val	missense
XL102	Male	COL4A5	21	hemi	c.1396G>A	p.Gly466Arg	missense
XL103	Male	COL4A5	21	hemi	c.1402_1403insTTTAC	p.Gln468LeufsTer8	small del/ins/dup
XL104	Male	COL4A5	21	hemi	c.1406G>A	p.Gly469Glu	missense
XL105	Male	COL4A5	21	hemi	c.1415G>A	p.Gly472Glu	missense
XL106	Male	COL4A5	21	hemi	c.1423+1del		splicing
XL107	Male	COL4A5	21	hemi	c.1424-4C>G		splicing
XL108	Male	COL4A5	22	hemi	c.1432G>A	p.Gly478Ser	missense
XL109	Male	COL4A5	22	hemi	c.1474_1475delinsTAT	p.Pro492TyrfsTer53	small del/ins/dup
XL110	Male	COL4A5	22	hemi	c.1480G>T	p.Gly494Cys	missense
XL111	Male	COL4A5	22	hemi	c.1516+3A>C		splicing
XL112	Female	COL4A5	23	het	c.1543G>A	p.Gly515Arg	missense

XL113	Male	COL4A5	23	hemi	c.1543G>A	p.Gly515Arg	missense
XL114	Male	COL4A5	23	hemi	c.1555C>T	p.Gln519Ter	nonsense
XL115	Male	COL4A5	23	hemi	c.1570G>C	p.Gly524Arg	missense
XL116	Male	COL4A5	23	hemi	c.1570G>T	p.Gly524Cys	missense
XL117	Female	COL4A5	24	het	c.1597G>A	p.Gly533Ar	missense
XL118	Female	COL4A5	24	het	c.1633G>T	p.Gly545Cys	missense
XL119	Female	COL4A5	24	het	c.1672G>A	p.Gly558Ser	missense
XL120	Male	COL4A5	24	hemi	c.1672G>A	p.Gly558Ser	missense
XL121	Female	COL4A5	24	het	c.1673G>T	p.Gly558Val	missense
XL122	Male	COL4A5	24	hemi	c.1673G>T	p.Gly558Val	missense
XL123	Female	COL4A5	24	het	c.1754G>A	p.Gly585Glu	missense
XL124	Male	COL4A5	24	hemi	c.1779+3G>T		splicing
XL125	Male	COL4A5	24	hemi	c.1780-2A>T		splicing
XL126	Female	COL4A5	25	het	c.1817G>A	p.Gly606Glu	missense
XL127	Female	COL4A5	25	het	c.1825G>C	p.Gly609Arg	missense
XL128	Male	COL4A5	25	hemi	c.1826G>T	p.Gly609Val	missense
XL129	Male	COL4A5	25	hemi	c.1843G>A	p.Gly615Arg	missense
XL130	Male	COL4A5	25	hemi	c.1870G>T	p.Gly624Cys	missense
XL131	Female	COL4A5	25	het	c.1871G>T	p.Gly624Val	missense
XL132	Female	COL4A5	25	het	c.1894G>A	p.Gly632Ser	missense
XL133	Male	COL4A5	25	hemi	c.1894G>A	p.Gly632Ser	missense
XL134	Female	COL4A5	25	het	c.1904G>A	p.Gly635Asp	missense
XL135	Female	COL4A5	25	het	c.1933C>T	p.Gln645Ter	nonsense

XL136	Female	COL4A5	26	het	c.2005G>A	p.Gly669Ser	missense
XL137	Male	COL4A5	26	hemi	c.2023G>A	p.Gly675Ser	missense
XL138	Male	COL4A5	26	hemi	c.2023G>A	p.Gly675Ser	missense
XL139	Male	COL4A5	26	hemi	c.2024G>A	p.Gly675Asp	missense
XL140	Male	COL4A5	26	hemi	c.2026G>A	p.Asp676Asn	missense
XL141	Female	COL4A5	26	het	c.2041G>C	p.Gly681Arg	missense
XL142	Male	COL4A5	27	hemi	c.2096G>A	p.Gly699Glu	missense
XL143	Female	COL4A5	27	het	c.2105G>A	p.Gly702Asp	missense
XL144	Male	COL4A5	27	hemi	c.2105G>A	p.Gly702Asp	missense
XL145	Male	COL4A5	27	hemi	c.2129G>A	p.Gly710Glu	missense
XL146	Male	COL4A5	27	hemi	c.2137G>A	p.Gly713Ser	missense
XL147	Male	COL4A5	27	hemi	c.2138G>T	p.Gly713Val	missense
XL148	Female	COL4A5	28	het	c.2155G>A	p.Gly719Arg	missense
XL149	Male	COL4A5	28	hemi	c.2165G>A	p.Gly722Glu	missense
XL150	Male	COL4A5	28	hemi	c.2227G>C	p.Gly743Arg	missense
XL151	Female	COL4A5	28	het	c.2236G>C	p.Gly746Arg	missense
XL152	Female	COL4A5	28	het	c.2244+1G>A		splicing
XL153	Male	COL4A5	29	hemi	c.2287G>A	p.Gly763Arg	missense
XL154	Male	COL4A5	29	hemi	c.2287G>A	p.Gly763Arg	missense
XL155	Male	COL4A5	29	hemi	c.2288_2308del	p.Gly763_Gly769del	small del/ins/dup
XL156	Male	COL4A5	29	hemi	c.2288G>A	p.Gly763Glu	missense
XL157	Male	COL4A5	29	hemi	c.2288G>A	p.Gly763Glu	missense
XL158	Male	COL4A5	29	hemi	c.2297G>A	p.Gly766Asp	missense

XL159	Male	COL4A5	29	hemi	c.2329C>A	p.Arg777Ser	missense
XL160	Male	COL4A5	29	hemi	c.2329C>A	p.Arg777Ser	missense
XL161	Female	COL4A5	29	het	c.2332G>C	p.Gly778Arg	missense
XL162	Male	COL4A5	29	hemi	c.2341G>C	p.Gly781Arg	missense
XL163	Female	COL4A5	29	het	c.2357C>A	p.Pro786Gln	missense
XL164	Male	COL4A5	29	hemi	c.2395+1G>A		splicing
XL165	Male	COL4A5	29	hemi	c.2395+3A>G		splicing
XL166	Male	COL4A5	30	hemi	c.2425_2428del	p.Pro809TrpfsTer9	small del/ins/dup
XL167	Female	COL4A5	30	het	c.2441G>A	p.Gly814Glu	missense
XL168	Male	COL4A5	30	hemi	c.2441G>A	p.Gly814Glu	missense
XL169	Female	COL4A5	30	het	c.2464G>C	p.Gly822Arg	missense
XL170	Male	COL4A5	30	hemi	c.2465G>A	p.Gly822Glu	missense
XL171	Male	COL4A5	30	hemi	c.2465G>T	p.Gly822Val	missense
XL172	Male	COL4A5	30	hemi	c.2501G>T	p.Gly834Val	missense
XL173	Male	COL4A5	31	hemi	c.2578G>A	p.Gly860Ser	missense
XL174	Male	COL4A5	31	hemi	c.2587G>A	p.Gly863Ser	missense
XL175	Female	COL4A5	31	het	c.2605G>A	p.Gly869Arg	missense
XL176	Female	COL4A5	31	het	c.2605G>A	p.Gly869Arg	missense
XL177	Male	COL4A5	31	hemi	c.2605G>A	p.Gly869Arg	missense
XL178	Male	COL4A5	31	hemi	c.2605G>A	p.Gly869Arg	missense
XL179	Male	COL4A5	31	hemi	c.2623G>A	p.Gly875Arg	missense
XL180	Male	COL4A5	31	hemi	c.2624G>A	p.Gly875Glu	missense
XL181	Male	COL4A5	31	hemi	c.2633G>A	p.Gly878Glu	missense

XL182	Male	COL4A5	31	hemi	c.2659G>T	p.Gly887Cys	missense
XL183	Male	COL4A5	31	hemi	c.2660G>C	p.Gly887Ala	missense
XL184	Male	COL4A5	31	hemi	c.2677G>A	p.Gly893Ser	missense
XL185	Male	COL4A5	32	hemi	c.2678G>A	p.Gly893Asp	missense
XL186	Female	COL4A5	32	het	c.2687del	p.Gly896ValfsTer5	small del/ins/dup
XL187	Male	COL4A5	32	hemi	c.2687G>C	p.Gly896Ala	missense
XL188	Male	COL4A5	32	hemi	c.2719C>T	p.Pro907Ser	missense
XL189	Male	COL4A5	32	hemi	c.2722G>T	p.Gly908Ter	nonsense
XL190	Male	COL4A5	32	hemi	c.2759G>T	p.Gly920Val	missense
XL191	Male	COL4A5	32	hemi	c.2767+4A>G		splicing
XL192	Male	COL4A5	33	hemi	c.2776G>T	p.Gly926Cys	missense
XL193	Male	COL4A5	33	hemi	c.2795G>T	p.Gly932Val	missense
XL194	Female	COL4A5	33	het	c.2830G>A	p.Gly944Arg	missense
XL195	Male	COL4A5	33	hemi	c.2840G>A	p.Gly947Asp	missense
XL196	Male	COL4A5	33	hemi	c.2840G>A	p.Gly947Asp	missense
XL197	Male	COL4A5	33	hemi	c.2882del	p.Gly961AlafsTer35	small del/ins/dup
XL198	Female	COL4A5	33	het	c.2902dup	p.Glu968GlyfsTer43	small del/ins/dup
XL199	Male	COL4A5	34	hemi	c.2999G>T	p.Gly1000Val	missense
XL200	Female	COL4A5	34	het	c.3016+1G>T		splicing
XL201	Male	COL4A5	34	hemi	c.3016+2T>C		splicing
XL202	Male	COL4A5	35	hemi	c.3035G>A	p.Gly1012Asp	missense
XL203	Female	COL4A5	35	het	c.3044G>A	p.Gly1015Glu	missense
XL204	Male	COL4A5	35	hemi	c.3053del	p.Gly1018ValfsTer3	small del/ins/dup

XL205	Male	COL4A5	35	hemi	c.3088G>A	p.Gly1030Ser	missense
XL206	Male	COL4A5	35	hemi	c.3088G>A	p.Gly1030Ser	missense
XL207	Male	COL4A5	35	hemi	c.3107-1G>A		splicing
XL208	Female	COL4A5	35	het	c.3107-2A>G		splicing
XL209	Female	COL4A5	36	het	c.3116_3121del	p.Gly1039_Val1040del	small del/ins/dup
XL210	Male	COL4A5	36	hemi	c.3154C>T	p.Gln1052Ter	nonsense
XL211	Male	COL4A5	36	hemi	c.3160G>A	p.Gly1054Ser	missense
XL212	Male	COL4A5	36	hemi	c.3160G>A	p.Gly1054Ser	missense
XL213	Female	COL4A5	36	het	c.3179G>A	p.Gly1060Glu	missense
XL214	Male	COL4A5	36	hemi	c.3179G>A	p.Gly1060Glu	missense
XL215	Female	COL4A5	36	het	c.3196G>A	p.Gly1066Ser	missense
XL216	Male	COL4A5	36	hemi	c.3196G>A	p.Gly1066Ser	missense
XL217	Female	COL4A5	36	het	c.3206G>T	p.Gly1069Val	missense
XL218	Female	COL4A5	36	het	c.3246+3A>C		splicing
XL219	Male	COL4A5	36	hemi	c.3247-1G>A		splicing
XL220	Male	COL4A5	37	hemi	c.3293G>T	p.Gly1098Val	missense
XL221	Male	COL4A5	37	hemi	c.3310G>C	p.Gly1104Arg	missense
XL222	Female	COL4A5	37	het	c.3319G>A	p.Gly1107Arg	missense
XL223	Male	COL4A5	37	hemi	c.3319G>A	p.Gly1107Arg	missense
XL224	Male	COL4A5	37	hemi	c.3319G>A	p.Gly1107Arg	missense
XL225	Male	COL4A5	37	hemi	c.3320G>T	p.Gly1107Val	missense
XL226	Male	COL4A5	37	hemi	c.3346G>A	p.Gly1116Arg	missense
XL227	Female	COL4A5	37	het	c.3347G>T	p.Gly1116Val	missense

XL228	Male	COL4A5	37	hemi	c.3353dup	p.Gly1119ArgfsTer18	small del/ins/dup
XL229	Female	COL4A5	37	het	c.3373G>A	p.Gly1125Arg	missense
XL230	Male	COL4A5	37	hemi	c.3373G>A	p.Gly1125Arg	missense
XL231	Male	COL4A5	38	hemi	c.3399del	p.Gly1134ValfsTer18	small del/ins/dup
XL232	Male	COL4A5	38	hemi	c.3427G>A	p.Gly1143Ser	missense
XL233	Male	COL4A5	38	hemi	c.3427G>A	p.Gly1143Ser	missense
XL234	Female	COL4A5	38	het	c.3455-9A>G		splicing
XL235	Male	COL4A5	38	hemi	c.3455-9A>G		splicing
XL236	Male	COL4A5	39	hemi	c.3462_3474del	p.Gly1155AsnfsTer146	small del/ins/dup
XL237	Male	COL4A5	39	hemi	c.3499G>A	p.Gly1167Ser	missense
XL238	Male	COL4A5	39	hemi	c.3499G>A	p.Gly1167Ser	missense
XL239	Female	COL4A5	39	het	c.3500G>A	p.Gly1167Asp	missense
XL240	Female	COL4A5	39	het	c.3508G>A	p.Gly1170Ser	missense
XL241	Male	COL4A5	39	hemi	c.3508G>A	p.Gly1170Ser	missense
XL242	Male	COL4A5	39	hemi	c.3508G>A	p.Gly1170Ser	missense
XL243	Male	COL4A5	39	hemi	c.3508G>A	p.Gly1170Ser	missense
XL244	Male	COL4A5	39	hemi	c.3508G>A	p.Gly1170Ser	missense
XL245	Male	COL4A5	39	hemi	c.3508G>A	p.Gly1170Ser	missense
XL246	Male	COL4A5	39	hemi	c.3509G>T	p.Gly1170Val	missense
XL247	Female	COL4A5	39	het	c.3520_3521dup	p.Pro1175PhefsTer131	small del/ins/dup
XL248	Male	COL4A5	39	hemi	c.3526G>C	p.Gly1176Arg	missense
XL249	Male	COL4A5	39	hemi	c.3535G>A	p.Gly1179Arg	missense
XL250	Male	COL4A5	40	hemi	c.3578del	p.Gly1193AspfsTer112	small del/ins/dup

XL251	Male	COL4A5	40	hemi	c.3587G>A	p.Gly1196Glu	missense
XL252	Female	COL4A5	40	het	c.3596G>A	p.Gly1199Glu	missense
XL253	Male	COL4A5	41	hemi	c.3614G>T	p.Gly1205Val	missense
XL254	Female	COL4A5	41	het	c.3617_3618del	p.Asp1206GlyfsTer45	small del/ins/dup
XL255	Male	COL4A5	41	hemi	c.3623G>A	p.Gly1208Glu	missense
XL256	Male	COL4A5	41	hemi	c.3668G>A	p.Gly1223Asp	missense
XL257	Male	COL4A5	41	hemi	c.3685G>A	p.Gly1229Ser	missense
XL258	Male	COL4A5	41	hemi	c.3685G>A	p.Gly1229Ser	missense
XL259	Male	COL4A5	41	hemi	c.3685G>A	p.Gly1229Ser	missense
XL260	Male	COL4A5	41	hemi	c.3685G>A	p.Gly1229Ser	missense
XL261	Male	COL4A5	41	hemi	c.3685G>A	p.Gly1229Ser	missense
XL262	Male	COL4A5	41	hemi	c.3685G>T	p.Gly1229Cys	missense
XL263	Male	COL4A5	41	hemi	c.3686G>A	p.Gly1229Asp	missense
XL264	Female	COL4A5	41	het	c.3703G>T	p.Gly1235Cys	missense
XL265	Male	COL4A5	41	hemi	c.3721G>T	p.Gly1241Cys	missense
XL266	Male	COL4A5	41	hemi	c.3731G>A	p.Gly1244Asp	missense
XL267	Male	COL4A5	41	hemi	c.3731G>A	p.Gly1244Asp	missense
XL268	Male	COL4A5	41	hemi	c.3733_3734delinsG	p.Pro1245GlufsTer60	small del/ins/dup
XL269	Female	COL4A5	41	het	c.3771A>T	p.Gln1257His	missense
XL270	Female	COL4A5	41	het	c.3785del	p.Arg1262AsnfsTer43	small del/ins/dup
XL271	Male	COL4A5	41	hemi	c.3790+1G>A		splicing
XL272	Female	COL4A5	44	het	c.3817G>A	p.Gly1273Ser	missense
XL273	Male	COL4A5	44	hemi	c.3817G>A	p.Gly1273Ser	missense

XL274	Male	COL4A5	44	hemi	c.3819_3863del	p.P1274_G1288del	small del/ins/dup
XL275	Male	COL4A5	44	hemi	c.3942+5G>A		splicing
XL276	Female	COL4A5	45	het	c.3958C>T	p.Pro1320Ser	missense
XL277	Male	COL4A5	45	hemi	c.4015G>A	p.Gly1339Ser	missense
XL278	Female	COL4A5	46	het	c.4060G>A	p.Gly1354Arg	missense
XL279	Male	COL4A5	46	hemi	c.4079G>T	p.Gly1360Val	missense
XL280	Male	COL4A5	46	hemi	c.4087+1G>A		splicing
XL281	Male	COL4A5	46	hemi	c.4088-2A>G		splicing
XL282	Male	COL4A5	47	hemi	c.4097G>T	p.Gly1366Val	missense
XL283	Male	COL4A5	47	hemi	c.4112del	p.Ser1371Ter	small del/ins/dup
XL284	Male	COL4A5	47	hemi	c.4172G>A	p.Gly1391Glu	missense
XL285	Male	COL4A5	47	hemi	c.4217-2A>G		splicing
XL286	Female	COL4A5	48	het	c.4242dup	p.Gly1415TrpfsTer9	small del/ins/dup
XL287	Female	COL4A5	48	het	c.4244G>A	p.Gly1415Glu	missense
XL288	Female	COL4A5	48	het	c.4273del	p.Ala1425GlnfsTer129	small del/ins/dup
XL289	Male	COL4A5	48	hemi	c.4295del	p.Pro1432GlnfsTer122	small del/ins/dup
XL290	Male	COL4A5	48	hemi	c.4297G>A	p.Gly1433Ser	missense
XL291	Male	COL4A5	48	hemi	c.4297G>A	p.Gly1433Ser	missense
XL292	Male	COL4A5	48	hemi	c.4297G>A	p.Gly1433Ser	missense
XL293	Male	COL4A5	48	hemi	c.4297G>A	p.Gly1433Ser	missense
XL294	Male	COL4A5	48	hemi	c.4309C>T	p.Gln1437Ter	nonsense
XL295	Female	COL4A5	48	het	c.4315+1G>T		splicing
XL296	Male	COL4A5	48	hemi	c.4315+1G>T		splicing

XL297	Male	COL4A5	48	hemi	c.4316-1G>A		splicing
XL298	Male	COL4A5	48	hemi	c.4316-5_4316del		small del/ins/dup
XL299	Female	COL4A5	49	het	c.4316G>T	p.Gly1439Val	missense
XL300	Female	COL4A5	49	het	c.4324G>A	p.Gly1442Ser	missense
XL301	Female	COL4A5	49	het	c.4342G>A	p.Gly1448Ser	missense
XL302	Male	COL4A5	49	hemi	c.4352G>A	p.Gly1451Glu	missense
XL303	Female	COL4A5	49	het	c.4360G>A	p.Gly1454Ser	missense
XL304	Male	COL4A5	49	hemi	c.4360G>C	p.Gly1454Arg	missense
XL305	Male	COL4A5	49	hemi	c.4370G>A	p.Gly1457Asp	missense
XL306	Female	COL4A5	49	het	c.4385_4398delinsA	p.Ser1462Ter	small del/ins/dup
XL307	Female	COL4A5	49	het	c.4456_4461del	p.Thr1486_Leu1487del	small del/ins/dup
XL308	Male	COL4A5	49	hemi	c.4457C>G	p.Thr1486Arg	missense
XL309	Male	COL4A5	49	hemi	c.4457C>G	p.Thr1486Arg	missense
XL310	Male	COL4A5	49	hemi	c.4457C>G	p.Thr1486Arg	missense
XL311	Male	COL4A5	49	hemi	c.4457C>G	p.Thr1486Arg	missense
XL312	Male	COL4A5	49	hemi	c.4457C>G	p.Thr1486Arg	missense
XL313	Female	COL4A5	49	het	c.4491T>A	p.Tyr1497Ter	nonsense
XL314	Female	COL4A5	49	het	c.4495C>T	p.Gln1499Ter	nonsense
XL315	Female	COL4A5	49	het	c.4510G>A	p.Ala1504Thr	missense
XL316	Female	COL4A5	49	het	c.4519C>T	p.Gln1507Ter	nonsense
XL317	Female	COL4A5	49	het	c.4528G>A	p.Gly1510Arg	missense
XL318	Male	COL4A5	49	hemi	c.4528G>A	p.Gly1510Arg	missense
XL319	Male	COL4A5	50	hemi	c.4540_4545del	p.Ser1514_Cys1515del	small del/ins/dup

XL320	Male	COL4A5	50	hemi	c.4623T>A	p.Tyr1541Ter	nonsense
XL321	Male	COL4A5	50	hemi	c.4632G>T	p.Trp1544Cys	missense
XL322	Male	COL4A5	50	hemi	c.4681del	p.Gln1561ArgfsTer19	small del/ins/dup
XL323	Female	COL4A5	50	het	c.4689_4699delinsTGATTTAGCTGTGACTTTTGATTTAGTGTCACAGCTAAATCAAATGTCAGTGAGCATTGAT	p.Gln1564AspfsTer33	small del/ins/dup
XL324	Female	COL4A5	50	het	c.4705C>T	p.Arg1569Ter	nonsense
XL325	Female	COL4A5	50	het	c.4706+1G>A		splicing
XL326	Male	COL4A5	51	hemi	c.4709G>A	p.Cys1570Tyr	missense
XL327	Male	COL4A5	51	hemi	c.4718G>T	p.Cys1573Phe	missense
XL328	Female	COL4A5	51	het	c.4724C>A	p.Ala1575Asp	missense
XL329	Female	COL4A5	51	het	c.4768C>A	p.Pro1590Thr	missense
XL330	Male	COL4A5	51	hemi	c.4768C>A	p.Pro1590Thr	missense
XL331	Female	COL4A5	51	het	c.4800G>T	p.Trp1600Cys	missense
XL332	Female	COL4A5	52	het	c.4880T>C	p.Leu1627Ser	missense
XL333	Male	COL4A5	52	hemi	c.4909G>T	p.Glu1637Ter	nonsense
XL334	Male	COL4A5	52	hemi	c.4928C>T	p.Thr1643Ile	missense
XL335	Female	COL4A5	52	het	c.4931_4932dup	p.Asn1645ValfsTer15	small del/ins/dup
XL336	Male	COL4A5	52	hemi	c.4931G>T	p.Cys1644Phe	missense
XL337	Male	COL4A5	52	hemi	c.4946A>G	p.Asn1649Ser	missense
XL338	Male	COL4A5	52	hemi	c.4980del	p.Ser1661GlnfsTer12	small del/ins/dup
XL339	Male	COL4A5	52	hemi	c.4994G>A	p.Ser1665Asn	missense
XL340	Male	COL4A5	53	hemi	c.4995-2A>T		splicing
XL341	Female	COL4A5	53	het	c.5038C>T	p.Arg1680Ter	nonsense

XL342	Female	COL4A5	53	het	c.5047C>T	p.Arg1683Ter	nonsense
XL343	Male	COL4A5	53	hemi	c.5048G>A	p.Arg1683Gln	missense
XL344	Male	COL4A5	53	hemi	c.5048G>A	p.Arg1683Gln	missense
XL345	Male	COL4A5	53	hemi	c.5048G>A	p.Arg1683Gln	missense
XL346	Male	COL4A5	53	hemi	c.5048G>C	p.Arg1683Pro	missense
XL347	Male	COL4A5	53	hemi	c.5050T>C	p.Cys1684Arg	missense
XL348	Female	COL4A5	53	het	c.5053C>T	p.Gln1685Ter	nonsense
XL349	Female	COL4A5	53	het	c.5053C>T	p.Gln1685Ter	nonsense
XL350	Female	COL4A5	53	het	c.5053C>T	p.Gln1685Ter	nonsense
XL351	Female	COL4A5		het	ex11-36 dup		CNV
XL352	Male	COL4A5		hemi	ex1-3 del		CNV
XL353	Male	COL4A5		hemi	ex2 del		CNV
XL354	Male	COL4A5		hemi	ex29 del		CNV
XL355	Female	COL4A5		het	ex34-36 del		CNV
XL356	Male	COL4A5		hemi	ex37-41 dup		CNV
XL357	Male	COL4A5		hemi	ex37-47 del		CNV
XL358	Male	COL4A5		hemi	ex38-44 del		CNV
XL359	Male	COL4A5		hemi	ex41-48 dup		CNV
XL360	Female	COL4A5		het	ex4-18 del		CNV
XL361	Male	COL4A5		hemi	ex44 del		CNV
XL362	Male	COL4A5		hemi	ex5-36 dup		CNV
XL363	Male	COL4A5		hemi	ex5-36 dup		CNV

[a]The transcript version is NM_033380.
Abbreviation: del, deletion; ins,insertion; dup, duplication; ex, exon; CNV, copy number variation; hemi, hemizygous; het, heterozygous

STROBE Statement—Checklist of items that should be included in reports of ***cohort studies***

	Item No	Recommendation	Page No
**Title and abstract**	1	(*a*) Indicate the study's design with a commonly used term in the title or the abstract	Page 1-2
		(*b*) Provide in the abstract an informative and balanced summary of what was done and what was found	Page 3-4
**Introduction**			
Background/rationale	2	Explain the scientific background and rationale for the investigation being reported	Page 5-6
Objectives	3	State specific objectives, including any prespecified hypotheses	Page 6
**Methods**			
Study design	4	Present key elements of study design early in the paper	Page 7
Setting	5	Describe the setting, locations, and relevant dates, including periods of recruitment, exposure, follow-up, and data collection	Page 7-9
Participants	6	(*a*) Give the eligibility criteria, and the sources and methods of selection of participants. Describe methods of follow-up	Page 7
		(*b*) For matched studies, give matching criteria and number of exposed and unexposed	N/A
Variables	7	Clearly define all outcomes, exposures, predictors, potential confounders, and effect modifiers. Give diagnostic criteria, if applicable	Page 8-10
Data sources/ measurement	8*	For each variable of interest, give sources of data and details of methods of assessment (measurement). Describe comparability of assessment methods if there is more than one group	Page 8-9
Bias	9	Describe any efforts to address potential sources of bias	Page 8-9
Study size	10	Explain how the study size was arrived at	Page 7-8
Quantitative variables	11	Explain how quantitative variables were handled in the analyses. If applicable, describe which groupings were chosen and why	Page 9
Statistical methods	12	(*a*) Describe all statistical methods, including those used to control for confounding	Page 10-12
		(*b*) Describe any methods used to examine subgroups and interactions	Page 10-11
		(*c*) Explain how missing data were addressed	N/A
		(*d*) If applicable, explain how loss to follow-up was addressed	N/A
		(*e*) Describe any sensitivity analyses	Page 11
**Results**			
Participants	13*	(a) Report numbers of individuals at each stage of study—eg numbers potentially eligible, examined for eligibility, confirmed eligible, included in the study, completing follow-up, and analysed	Table S1
		(b) Give reasons for non-participation at each stage	N/A
		(c) Consider use of a flow diagram	N/A
Descriptive data	14*	(a) Give characteristics of study participants (eg demographic, clinical, social) and information on exposures and potential confounders	Page 11 Table 1
		(b) Indicate number of participants with missing data for each variable of interest	Table S2
		(c) Summarise follow-up time (eg, average and total amount)	Page 12
Outcome data	15*	Report numbers of outcome events or summary measures over time	Page 12 Table S1
Main results	16	(*a*) Give unadjusted estimates and, if applicable, confounder-adjusted estimates and their precision (eg, 95% confidence interval). Make clear which confounders were adjusted for and why they were included	

		(*b*) Report category boundaries when continuous variables were categorized<br>(*c*) If relevant, consider translating estimates of relative risk into absolute risk for a meaningful time period	Table S2
Other analyses	17	Report other analyses done—eg analyses of subgroups and interactions, and sensitivity analyses	Page 14-16
**Discussion**			
Key results	18	Summarise key results with reference to study objectives	Page 17
Limitations	19	Discuss limitations of the study, taking into account sources of potential bias or imprecision. Discuss both direction and magnitude of any potential bias	Page 20
Interpretation	20	Give a cautious overall interpretation of results considering objectives, limitations, multiplicity of analyses, results from similar studies, and other relevant evidence	Page 18-20
Generalisability	21	Discuss the generalisability (external validity) of the study results	Page 19-20
**Other information**			
Funding	22	Give the source of funding and the role of the funders for the present study and, if applicable, for the original study on which the present article is based	Page 20-21

*Give information separately for exposed and unexposed groups.

Note: An Explanation and Elaboration article discusses each checklist item and gives methodological background and published examples of transparent reporting. The STROBE checklist is best used in conjunction with this article (freely available on the Web sites of PLoS Medicine at http://www.plosmedicine.org/, Annals of Internal Medicine at http://www.annals.org/, and Epidemiology at http://www.epidem.com/). Information on the STROBE Initiative is available at http://www.strobe-statement.org.
